# Audio-visual integration in noise: Influence of auditory and visual stimulus degradation on eye movements and perception of the McGurk effect

**DOI:** 10.3758/s13414-020-02042-x

**Published:** 2020-06-12

**Authors:** Jemaine E. Stacey, Christina J. Howard, Suvobrata Mitra, Paula C. Stacey

**Affiliations:** 1 Department of Psychology, Nottingham Trent University, Nottingham, NG1 4BU UK; 2 National Institute for Health Research, Nottingham Biomedical Research Centre, Nottingham, NG1 5DU UK

**Keywords:** McGurk effect, Eye movements, Multisensory perception, Audio-visual integration

## Abstract

Seeing a talker’s face can aid audiovisual (AV) integration when speech is presented in noise. However, few studies have simultaneously manipulated auditory and visual degradation. We aimed to establish how degrading the auditory and visual signal affected AV integration. Where people look on the face in this context is also of interest; Buchan, Paré and Munhall (*Brain Research*, *1242*, 162–171, 2008) found fixations on the mouth increased in the presence of auditory noise whilst Wilson, Alsius, Paré and Munhall (*Journal of Speech, Language, and Hearing Research*, *59*(4), 601–615, 2016) found mouth fixations decreased with decreasing visual resolution. In Condition 1, participants listened to clear speech, and in Condition 2, participants listened to vocoded speech designed to simulate the information provided by a cochlear implant. Speech was presented in three levels of auditory noise and three levels of visual blurring. Adding noise to the auditory signal increased McGurk responses, while blurring the visual signal decreased McGurk responses. Participants fixated the mouth more on trials when the McGurk effect was perceived. Adding auditory noise led to people fixating the mouth more, while visual degradation led to people fixating the mouth less. Combined, the results suggest that modality preference and where people look during AV integration of incongruent syllables varies according to the quality of information available.

**Electronic supplementary material:**

The online version of this article (10.3758/s13414-020-02042-x) contains supplementary material, which is available to authorized users.

## Introduction

In our everyday environment we are bombarded with information from our senses; multisensory integration is essential for helping to consolidate information and make sense of the world. Multisensory information is often complementary; for example, to understand the person speaking during a conservation, the auditory element (the voice of the speaker) and the visual element (the face of the speaker) are combined into a single percept. It has been suggested that this occurs because sensory pathways in the brain are cross-modal, meaning they can be influenced by other modalities (Shimojo & Shams, 2001). This idea is underpinned in part by evidence from audiovisual perceptual illusions that arise when synchronized, incongruent information is presented in the auditory and visual modalities. Research has shown that auditory stimuli can influence visual perception, as demonstrated in the sound-induced flash illusion in which viewers perceive a unitary flash as a double flash if it coincides with two auditory beeps (Shams, Kamitani & Shimojo, 2000). Two flashes can also be perceived as a single flash if a single beep is presented; this is termed the fusion effect (Andersen, Tiippana & Sams, 2004).

One illusion that exemplifies the influence of visual information is the *McGurk effect*, which is also widely used as a measure of AV integration. The McGurk effect occurs when incongruent auditory and visual syllables are presented simultaneously (McGurk & McDonald, 1976), resulting in an illusory auditory percept. For example, hearing a voice say /ba/ and seeing a face say /ga/ has the effect that listeners often hear a different syllable to that of the voice, for example /da/ or /tha/. Not everyone perceives the McGurk effect, however, and despite extensive study, the prevalence of the McGurk effect is difficult to determine. A recent review (Alsius, Paré & Munhall, 2017) reported that the proportion of McGurk responses ranged from 0.32 to 0.98. While there is some evidence that perception of the McGurk effect is correlated with the amount of visual enhancement people experience when listening to sentences (Grant & Seitz, 1998), it is important to note that the validity of the McGurk effect has been called in to question in recent years (Alsius et al., 2017; Rosenblum, 2019; Van Engen, Xie & Chandrasekaran, 2017). This is due to evidence that the McGurk effect does not correlate with other measures of AV integration (Van Engen et al., 2017). Despite this, degrading McGurk stimuli and using eye-movement measures can still tell us about how visual information is used when speech is degraded in noise. Studying factors that can influence the perception of AV illusions is useful for understanding how our senses interact. In their review, Shams and Kim (2010) point out that, traditionally, vision was viewed as the dominant sense. However, this is context dependent and either audition or vision can dominate depending on the demands of the task. Robinson, Chandra and Scinnett (2016) found that increasing response options resulted in a switch to visual dominance, suggesting that sensory dominance is modulated by attention. Visual dominance has also been found to increase across the life span (Hirst, Stacey, Cragg, Stacey & Allen, 2018).

Furthermore, auditory or visual dominance can depend on the weighted reliability of information from each sense (Ernst & Bülthoff, 2004; Witten & Knudsen, 2005). When faced with the task of understanding speech in quiet listening conditions, audition is usually the dominant sense as speech can be easily identified from auditory information alone (Gatehouse & Gordon, 1990; Shannon, Zeng, Kamath, Wygonski & Ekelid, 1995). In contrast, it is very difficult to understand speech from visual information only (lipreading; Bernstein & Liebenthal, 2014). However, for AV speech perception, if information in one modality is degraded this can shift sensory dominance to the more reliable sense and in turn influence AV integration. For example, trying to understand someone speaking in a noisy room may result in more reliance on the visual information; Ma, Zhou, Ross, Foxe and Parra (2009) found that visual enhancement for AV words occurred at signal-to noise ratios (SNRs) of -8 dB or above. According to the Principle of Inverse Effectiveness (Meredith & Stein, 1986), when unisensory information is degraded, AV integration improves. This suggests that visual information would be of most benefit when auditory information is severely degraded by noise. Ross, Saint-Amour, Leavitt, Javitt and Foxe (2007) also found that visual enhancement peaked at -12 dB. This suggests that there is an optimum level of auditory noise at which visual information improves speech perception. However, Tye-Murray, Sommers, Spehar, Myerson and Hale (2010) reduced the clarity of both the auditory and visual signal by using SNRs and lowering the contrast of the image. They found that reducing the quality of information in either modality did not enhance AV integration. As the McGurk effect is dependent on the visual signal, it is expected that auditory noise would result in more reliance on the visual signal, which would enhance the illusion. This is supported by studies that show that when incongruent McGurk syllables were presented in white noise the McGurk effect increased (Hirst et al., 2018; Sekiyama, Soshi & Sakamoto, 2014).

As well as exploring listening contexts that simulate hearing impairments, studies have investigated what happens when the visual signal is degraded to better understand the benefit of visual information. Research finds that degrading the visual signal decreases the McGurk effect but does not inhibit it completely (Paré, Richler, ten Hove & Munhall, 2003; Wilson, Alsius, Paré & Munhall, 2016). MacDonald, Andersen and Bachmann (2000) found that as pixelation of the faces increased, fewer instances of the McGurk effect were reported (MacDonald et al., 2000). Similarly, when facial resolution was manipulated, the McGurk effect increased with increasing visual resolution and was less affected by the removal of high-frequency information (Wilson et al., 2016). Tye-Murray, Spehar, Myerson, Hale and Sommers (2016a) degraded the auditory signal with multi-talker babble and blurred the visual signal. They found that a degraded visual signal reduced performance on a task in which participants had to identify target words to complete sentences.

An additional form of auditory degradation is that experienced by people with hearing impairments. People with profound deafness can have their hearing partially restored by cochlear implants (CIs); however, CIs do not restore normal hearing but deliver a signal that is temporally and spectrally degraded, meaning they often struggle to understand speech in noise. Research with CI users suggests they benefit from visual information and may be more adept at AV integration compared to people with normal hearing (Rouger et al., 2007). In conjunction with this, CI users perceive the McGurk effect more often compared to normal hearing listeners (Stropahl, Schellhardt, and Debener, 2017). This benefit of visual information and increased perception of the McGurk effect could be due to CI users’ tendency to look at the mouth more compared to people with normal hearing (Mastrantuono, Saldaña & Rodríguez-Ortiz, 2017). People with CIs might look at the mouth more in order to help them get more information from the visual signal, in the face of auditory degradation. This can be tested in normal-hearing listeners by using *vocoded* speech (Shannon et al., 1995), which simulates the speech processing involved in a CI. Vocoding degrades the speech in two ways: (1) there is extensive blurring of the frequency information presented, and (2) rapid fluctuations in amplitude over time are removed. This impairs the understanding of speech in quiet and in noisy environments (e.g. Qin and Oxenham, 2003).

### Eye movements and audiovisual integration

Where people look on a talking face may be an important factor in explaining variability in AV integration in different situations and across individuals. Gurler, Doyle, Walker, Magnotti and Beauchamp (2015) divided participants into strong and weak perceivers of the McGurk effect; strong perceivers experienced the illusion on 50% or trials or more, weak perceivers in less than 50% of trials. They found that strong perceivers of the McGurk effect spent longer fixating the mouth than weak perceivers. Moreover, there was a correlation between McGurk effect perception and time spent fixating the mouth (Gurler et al., 2015). In contrast, however, Paré et al. (2003) found that fixating the mouth did not predict the extent to which the McGurk effect was perceived. When participants’ gaze was directed 20° away from the mouth, the McGurk effect was still present, suggesting that fixating the mouth is not always necessary to perceive the McGurk effect (Paré et al., 2003). This finding suggests that face movements that can be seen in peripheral vision are sufficient to produce the McGurk effect.

Gurler et al. (2015) suggested that the contradictory findings may be due to the pre-stimulus fixation-cross positioning, as their study used a peripheral fixation cross that appeared in one of four corners of the screen, whereas Paré et al. (2003) used a central fixation cross. The authors argue that the pre-stimulus peripheral fixation cross forces participants to make a planned eye movement to a particular part of the face, whereas a central fixation cross encourages participants to fixate centrally and attend to other parts of the face in the peripheral vision (Gurler et al., 2015). Arizpe, Kravitz, Yovel and Baker (2012) used a face recognition task and varied the location of starting fixations when participants viewed faces. They found that saccade latencies were influenced by the location of the starting fixation and that central fixations resulted in ‘longer saccade latencies’ than peripheral fixations. Similarly, Hoffman and Subramaniam (1995) looked at how eye movements influence target detection and found that when targets were presented randomly in one of the four corners of the screen, making a saccade to the location of the target increased successful target detection compared to when targets were attended in peripheral vision.

Fixating directly on the mouth and surrounding area may be particularly important when the auditory signal is degraded as this would enable extraction of better quality visual information to enable AV integration. When monologues were presented in high levels of background noise including music and multilingual talkers, participants looked at the eyes approximately half of the time (Vatikiotis-Bateson, Eigsti, Yano & Munhall, 1998). It could be argued that this is due to the nature and length of the stimuli (45 s) as participants may be looking for social/emotional cues whilst listening to the narrative (Alsius, Wayne, Paré & Munhall, 2016). Another study found that participants focused more on the nose and mouth when sentences were presented in noise (multi-talker babble), again suggesting that the area directly surrounding the mouth is important (Buchan, Paré & Munhall, 2008). In the no-noise condition when a different talker spoke on every trial, participants focused on the mouth more compared to when the talker was consistent across trials, suggesting talker identity influences where people look (Buchan et al., 2008). Buchan et al. (2008) suggested this is consistent with a strategy in which viewers try to learn the identity of the talker by focussing on the mouth, as the physical attributes of the mouth may provide cues about the talker’s voice, which can aid AV integration.

### Current study

Collectively, these studies emphasise the importance of visual information for speech perception. What is unclear is how important fixating a talker’s mouth is for AV integration under degraded conditions. The present experiment aimed to clarify how perception of the McGurk effect and eye movements differ in background noise and using degraded auditory and visual stimuli. There were two separate conditions, the Clear Condition, which used undistorted ‘Clear’ speech, and a Vocoded Condition, which used ‘Vocoded’ speech to simulate the information provided by a CI. The overall aims were: (1) to replicate previous research by investigating how sensory AV integration changes when speech is subject to both auditory and visual degradation, (2) to explore eye movements in different levels and types of auditory noise (white noise and vocoded speech) and visual blur, and (3) to include the manipulation of fixation-cross position as this could have an influence on where people fixate on a face. This could account for some of the inconsistency in the literature in terms of whether fixating the mouth is important. Whilst a handful of studies have simultaneously manipulated the quality of the auditory and visual information (Alsius et al., 2016; McGettigan et al., 2012; Munhall, Kroos, Jozan, & Vatikiotis-Bateson, 2004; Tye-Murray, Spehar, Myerson et al., 2016a), this study provides a novel contribution in several ways. Firstly, different types of auditory noise were used with eye-tracking methods – to our knowledge this is the first paper to use vocoded speech presented in white noise to degrade McGurk stimuli and measure eye movements. Secondly, there is disparity in the literature as to whether looking at the mouth of the talker is necessary for the McGurk effect: Gurler et al. (2015) hypothesized that fixation-cross position might influence where people look on a face – our study is the first to test this hypothesis, which is important for informing future methods as where people look on a face may influence the quality of visual information received.

We predicted that McGurk responses would increase in auditory noise due to increased influence of the visual modality, but they would decrease in visual blur. As previous research shows that removing high spatial frequency visual information is not detrimental to McGurk-effect perception, we predict that McGurk responses will be reported with some visual blur, but will decrease when visual information is severely degraded. Additionally, we predict that the McGurk effect will be more likely to be perceived when participants were fixating the mouth, and this effect may be strongest when a peripheral fixation cross was used as participants are required to make an eye movement to task-relevant areas of the face such as the mouth. Following Gurler et al. (2015), we predict that stronger perceivers of the McGurk effect will look at the mouth more than weak perceivers. The results will establish how AV integration changes when information from both the auditory and visual senses is suboptimal. This potentially could also be used to aid people with hearing or visual impairments by creating training materials specifically aimed at developing strategies to improve AV integration.

## Clear condition

We used ‘Clear’ undistorted speech and investigated how AV integration and eye movements were affected by degrading the auditory and visual signal. To maintain consistency with other research (Gurler et al., 2015; Paré et al., 2003), a forced-choice task was used. We define a McGurk response as any non-auditory response to a McGurk stimulus.

### Method

#### Design

This experiment used a 3 × 3 × 2 mixed design. The within-subjects factors were Auditory Noise (No noise, Mid noise, High noise) and Visual Blur (No blur, Mid blur, High blur). The between-subjects factor was Fixation Cross position (Central or Peripheral). The dependent variable was McGurk effect perception, defined as responses participants made that correspond with the non-auditory signal. For the eye-movement analysis the key dependent variable of interest was the percentage of overall dwell time on the mouth. The dwell time measure includes all fixations and saccades that fall within an area of interest.

#### Participants

Participants were 37 students, five male and 32 female, aged from 19–48 years (M= 22.35), from Nottingham Trent University. A post hoc power analysis was conducted using a simulation-based method in R (R Core Team, 2017) to determine power with the sample size (N=31) used. Details of this analysis and the code are provided in the Online Supplementary Materials. Based on the effect sizes found in previous research that used a similar paradigm (Fixmer & Hawkins, 1998; Hirst et al., 2018), we specified medium to large effects and determined that if the effect were medium, power would be estimated at 0.97 for the logistic regression models and 0.98 for the linear random effects models, suggesting that the sample size used was sufficient. The project was approved by the Nottingham Trent University Social Sciences Research Ethics Committee. Students received course research credits for their time. All participants were native English speakers and had normal hearing and normal or corrected-to-normal vision. Participants also reported that they had not been diagnosed with any autism spectrum disorder (ASD) or dyslexia.

#### Stimuli and apparatus

There were four stimuli for each talker (one incongruent syllable + three congruent syllables), and four talkers provided the stimuli. There were three congruent syllables; /ba/, /da/ and /ga/ incongruent McGurk pairs were auditory /ba/ and visual /ga/ (ABVG). The four stimuli from each talker were presented in nine different conditions (visual blur: no blur, mid blur, high blur × auditory noise: none, mid, high). Each stimulus was presented twice, making a total of 144 trials (36 incongruent trials, 108 congruent trials).

Visual blur was created using Gaussian blurring at 40% and 60% in Premiere Pro v 9.0.0. White noise was created using Matlab (Mathworks, Natick, MA, USA) and added at two SNRs: -8 dB and -20 dB. Blur and noise levels were decided upon based on pilot testing; congruent stimuli (BA, GA, DA) were presented from the four talkers in nine separate levels of auditory noise and visual blur. Participants (N=10) were asked to report what syllable they perceived. The noise and blur levels at which correct responses decreased to approximately 50% were chosen to constitute the ‘high’ level of degradation. This was -20 dB for the auditory condition and 60% blur for the visual condition. The data point at approximately the middle of ceiling and poor performance was chosen to represent ‘mid’ noise. This was -8 dB for the auditory condition and 40% blur for the visual condition.

Stimuli were created by splicing together auditory and visual components using Adobe Premiere Pro. All stimuli were presented at the same sound level (average ~70 dB) determined by using a Svantek 977 sound-level meter combined with an artificial ear (Brüel & Kjær Type 4153). A 19-in. computer screen was used. Stimuli were presented via Experiment Centre and using HD280pro headphones (Sennheiser, Wedemark, Germany). Eye tracking was performed with a RED 500 SMI eye tracker and eye movements were recorded for the duration of each stimulus ~2,000 ms.

#### Procedure

Participants sat in front of a desk ~45 cm away from the eye tracker. Before the experiment began, participants were instructed to “watch and listen closely to the videos” whilst eye movements were recorded. A four-point calibration and validation procedure was performed before each participant began the experiment. Participants were required to watch videos of the talkers and then respond by repeating out loud what they heard from the following choices: /BA/, /GA/ /DA/ or /THA/. Responses were recorded using a Dictaphone. There were six practice trials, immediately after each video the four choices were displayed on the screen and participants were prompted to verbally state their choice. During the experimental trials all stimuli were displayed in a randomized order and a fixation cross was displayed. As soon as the participants made an eye movement to the fixation cross, this triggered the stimulus presentation. For half of the participants (N = 17) the fixation cross appeared in the centre of the screen and for the other half of the participants (N = 16) it appeared in one of four corners of the screen. The corner in which the fixation cross appeared was determined with 25% probability for each corner and randomised between trials.

#### Analyses

**Fig. 1:**
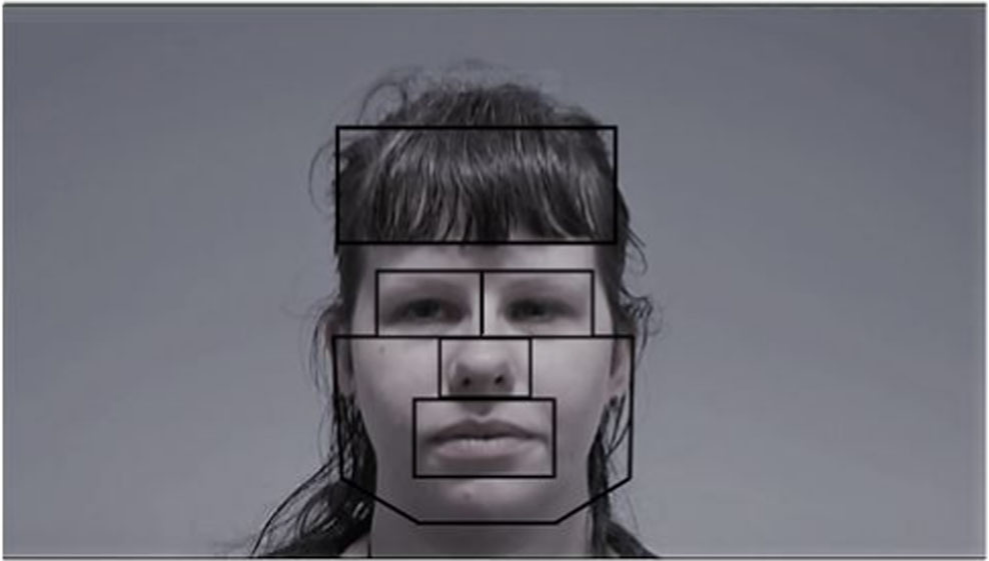
Six separate areas of interest were used encompassing the hair/forehead, the left and right eye, the chin/cheeks, nose and the mouth

The main statistical analyses were performed using multi-level models so that both participants and stimuli could be treated as random effects. Multi-level models avoid aggregating across stimuli, and are therefore less prone to Type 1 errors (Baguley, 2012). The random-effects structure included both random intercepts and random slopes. Model comparisons were carried out, and if interactions were not significant they were omitted. If convergence warnings occurred random effects were specified as independent (no correlations between random effects) and removed if they did not contribute to the model to prevent overfitting; this was determined if the variance was equal to zero (see Barr, Levy, Scheepers & Tily, 2013). If convergence warnings remained the optimizer was changed using control = lmerControl(optimizer = "Nelder_Mead"). For one model where a failure to converge was obtained we tested to see if the relative gradient value at which optimization stopped was sufficiently small. For this we executed (relgrad <- with(model@optinfo$derivs, solve(Hessian, gradient)), and ignored the convergence warning as max(abs(relgrad)) was smaller than 0.001. Error bars on figures represent 95% confidence intervals. To analyse the eye-tracking data, six main areas of interest (AOIs) were constructed, as shown in Fig. 1. The AOIs were the same size throughout the video and the mouth AOI was created so it covered the mouth aperture at its widest part.

### Results

Six participants were excluded after data collection and before analyses were conducted, four due to incomplete eye movement data, one because of a diagnosis of attention-deficit hyperactivity disorder (ADHD) and one because English was not their first language. Therefore, analyses were conducted with 31 participants.

#### Variability in McGurk effect perception across participants and stimuli

**Fig. 2:**
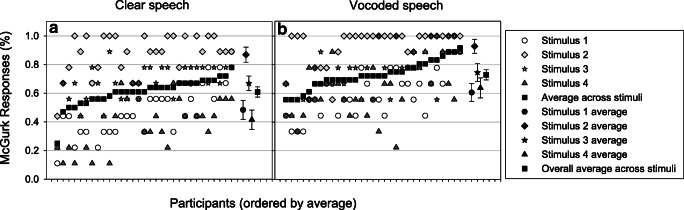
Variability in perception of the McGurk effect across participants and stimuli. Participants have been ordered according to their average across the four stimuli. Averages for each stimulus across participants are also shown. Panel **A** shows data for Clear speech and Panel **B** shows data for Vocoded speech

Perception of the McGurk effect varied across participants and stimuli, as shown in Fig. 2 (Panel A). Perception of the McGurk effect ranged from 25–78% (M= 60.8%, SD= 9.8%) across participants. Stimuli from different talkers also elicited the McGurk effect by different amounts – for example, the McGurk effect was perceived 86.8% (SD= 14.5%) of the time from Stimulus 2, but just 41.5% (SD= 18.1%) of the time from Stimulus 4.

#### Distribution of eye movements in each area of interest (AOI)

**Fig. 3:**
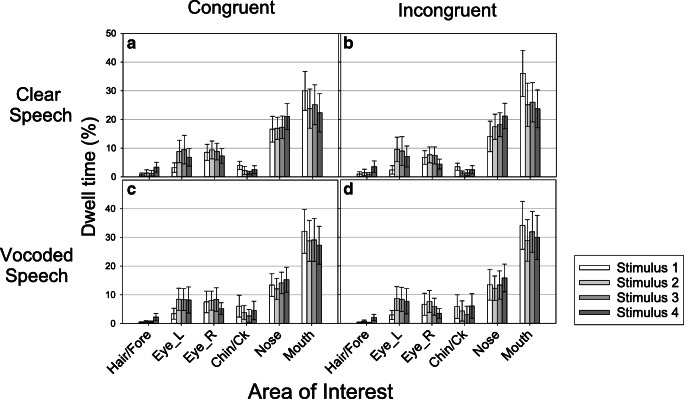
Percentage of dwell time in each area of interest according to Congruence and Stimulus. Panels **A** and **B** (Clear Condition) show data for Clear speech while Panels **C** and **D** (Vocoded Condition) show data for Vocoded speech

Figure 3 shows the distribution of eye movements across the different AOIs for each Talker. Panel A shows data for Congruent stimuli and Panel B shows data for Incongruent (McGurk) stimuli in the Clear condition. Broadly, the pattern of fixations was similar for the different talkers and across Congruent and Incongruent stimuli, with the mouth receiving the most dwell time (overall average 25.9%, SD 18.8%), followed by the nose (overall average 17.9%, SD 10.1%), followed by the eyes, then the hair/forehead and the chin/cheeks.

A 2 (Congruence) × 6 (AOI) × 4 (Stimulus) ANOVA confirmed that there were significant differences in dwell time according to AOI (F _5, 155_ = 29.59, p<0.001, ${\eta}_p^2$ = 0.49). There was additionally a significant interaction between Congruence and AOI (F _5, 155_ = 10.16, p<0.001, ${\eta}_p^2$ = 0.25). A comparison of the data in Fig. 3 Panels A and B shows that this was partly driven by dwell times on the mouth being longer for incongruent stimuli (M = 27.73%, SD = 19.51%) than for congruent stimuli (M =25.31, SD =18.65%; t (31) = 3.71, p<0.001). There were additionally significant interactions between AOI and Stimulus (F _15, 465_ = 10.52, p<0.001, ${\eta}_p^2$ = 0.25) and Congruence, AOI, and Stimulus (F _15, 465_ = 1.98, p = 0.015, ${\eta}_p^2$ = 0.06). As shown in Fig. 3, the overall pattern of fixations across the different talkers were broadly similar, but there were somewhat different patterns of fixations for the different talkers. For example, Talker 1 elicited more fixations on the mouth than the other stimuli, particularly when the stimuli were incongruent.

The following analyses include just the incongruent (McGurk) stimuli.

#### Effects of auditory noise and visual blur on McGurk responses

**Fig. 4:**
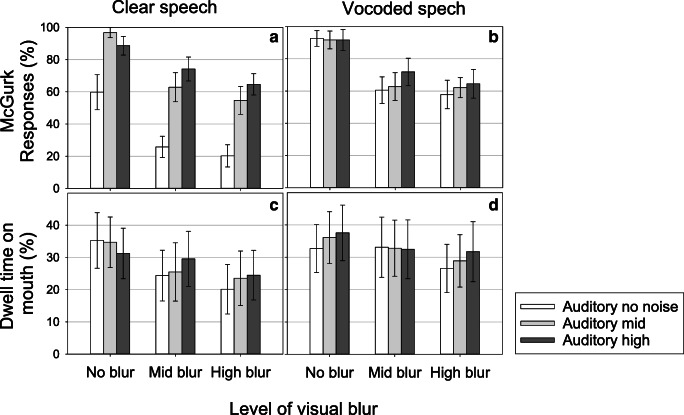
Effects of auditory and visual noise on the percentage of McGurk responses and the percentage dwell time on the mouth. Panels **A** and **B** show data from Clear speech and Panels **C** and **D** show data from Vocoded speech

The effects of auditory noise and visual blur on McGurk responses are shown in Fig. 4 (Panel A). McGurk responses were analysed using the Generalised Linear Model (glmer) function in R, carried out on whether participants perceived the McGurk effect on each trial according to Fixation-cross position, Auditory noise, and Visual blur. No interactions including Fixation cross were included as we did not expect Fixation cross to interact with Auditory noise or Visual blur. Including an interaction between Auditory and Visual noise did not significantly improve the model (ΔAIC = 1.9, Δ*X*^2^ = 0.15, p = 0.695). Interactions for random effects also did not significantly improve the model (ΔAIC = 5.4, Δ*X*^2^ = 10.57, p = 0.22), therefore all interactions were omitted. The estimated SD for the random effect of Participant was 0.53, and for Stimuli was 1.26. This therefore confirms that there was more variability associated with stimuli than with participants, and therefore that multi-level modelling is the appropriate statistical technique to use for these data. The results are presented in Table 1. There was no significant effect of Fixation cross, but there were significant effects of Auditory noise and Visual blur. As Fig. 4 (Panel A) shows, McGurk responses increased in the presence of auditory noise and decreased in the presence of visual blur.

**Table 1:** Clear speech: Multi-level modelling results from the analyses of the effect of Auditory noise, Visual blur, and Fixation cross on McGurk responses

Condition	B	SE	t-value	p-value
Intercept	0.85	0.74	1.14	0.253
Fixation cross	-0.02	0.24	-0.09	0.926
Auditory noise	1.20	0.20	5.88	<0.001
Visual blur	- 0.91	0.21	-4.32	<0.001

#### Effects of auditory noise and visual blur on dwell times on the mouth

The effects of auditory noise and visual blur on mouth dwell times are shown in Fig. 4 (Panel B). Statistical analysis was carried out using the Linear Model (lmer) function in R. This analysis looked at dwell time according to Fixation-cross position, Auditory noise, and Visual blur. No interaction with fixation cross was included (ΔAIC = 1.8, Δ*X*^2^ = 0.10, p = 0.74) or with Auditory noise and Visual blur as this did not significantly improve the model (ΔAIC = 1.2, Δ*X*^2^ = 3.10, p = 0.07); this was also the case for the interactions for random effects (ΔAIC = 7.7, Δ*X*^2^ = 8.31, p = 0.40).

The estimated SD for the random effect of Participant was 19.41, compared with an SD of 5.51 for Stimuli. This indicates that participants varied a great deal in their pattern of fixations, but there was less variation associated with the stimuli. Table 2 reports the model estimates from the full model and the associated p-values were obtained using Satterthwaite's method. There were no significant effects of Fixation cross or Auditory noise, but there was a significant main effect of Visual blur. As Fig. 4 (B) shows, dwell times on the mouth decreased with increasing visual blur.

**Table 2:** Clear speech: Multi-level modelling results from the analysis of the effect of Auditory noise, Visual blur, and Fixation cross on dwell time on mouth. Data were analysed using lmer, and significance was tested using Satterthwaite's method in R

Condition	b	SE	t-value	p-value
Intercept	20.09	11.61	1.73	0.093
Fixation cross	5.03	7.07	0.71	0.482
Auditory noise	0.74	0.64	1.16	0.255
Visual blur	-4.55	1.47	-3.08	0.015

.

#### Association between McGurk perception and dwell time on mouth, according to fixation-cross position

**Fig. 5:**
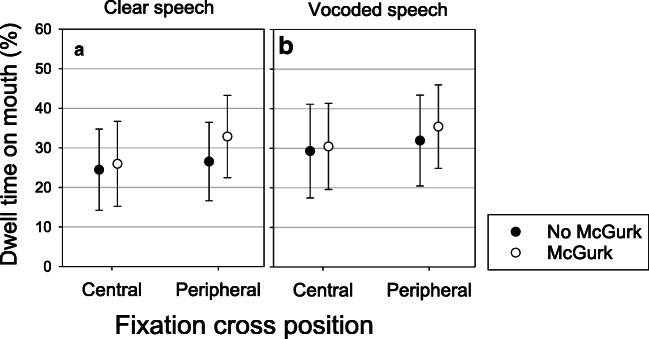
Percentage of dwell time on mouth according to fixation-cross position and whether the McGurk effect was perceived. Panel **A** shows data from Clear speech and Panel **B** shows data from Vocoded speech. Error bars show 95% confidence intervals

Figure 5 shows the percentage of time spent fixating the mouth according to whether or not the McGurk effect was perceived and the position of the fixation cross. This analysis was carried out on data for all levels of visual blur and auditory noise. The interaction between McGurk-effect perception and fixation-cross position was not significant and was dropped from the model (ΔAIC = 1.0, Δ*X*^2^ = 1.00, p = 0.31). Interactions for random effects resulted in high correlations and were therefore dropped from the final model. The estimated SD for the random effect of Participant was 19.40, compared with an SD of 6.00 for Stimuli, suggesting that there was variability in fixations on the mouth but less so for stimuli. More time was spent fixating the mouth when the McGurk effect was perceived (M= 34.20, SD = 28.94) than when it was not (M= 32.63, SD = 27.83). This difference was statistically significant (b = 6.10 (SE 1.36), t = 4.47, p < 0.001). There was no significant effect of Fixation cross (b = 5.04 (SE 7.08), t =0.712, p = 0.482).

#### Correlation between McGurk perception and dwell time on mouth

The average amount participants perceived the McGurk effect was calculated across stimuli for the non-degraded condition (auditory no-noise and visual no-blur). There was no significant correlation between the average amount the McGurk effect was perceived and the average time spent fixating the mouth (r _31_ = 0.092, p = 0.621).

### Discussion

We investigated how perception of the McGurk effect and accompanying eye movements were affected when speech was presented in auditory noise and visual blur. We found wide variability in perception of the McGurk effect across participants, ranging from 25–78%. Overall, McGurk responses were made 60.8% of the time. This supports previous findings that the McGurk effect is robust and that visual information influences auditory perception in context when people are presented with incongruent auditory and visual information (Campbell & Massaro, 1997; MacDonald et al., 2000; Thomas & Jordan, 2002). Interestingly, McGurk responses remain at around the 60% level when the auditory and visual signal is subject to the same level of degradation; visual no blur + auditory no noise = 60%, visual mid blur + auditory mid noise = 63%, visual high blur + auditory high noise = 65%. In terms of the effects of visual blur and auditory noise, our hypotheses were confirmed: McGurk effect perception increased in auditory noise and decreased in visual blur. Only when the auditory signal was presented without noise and the visual signal was blurred did McGurk responses fall to under 50%.

As expected, the majority of dwell time occurred on the mouth as that is where the speech information is predominantly provided. In addition, more time was spent fixating the mouth when stimuli were incongruent than when they were congruent, suggesting that participants directed their gaze to the mouth preferentially to resolve the conflict between the auditory and visual information presented. The second AOI most fixated was the nose, which provides a central location with which to view other features peripherally. Participants looked at the chin/cheek area the least, but still sometimes perceived the McGurk effect whilst fixating this area, suggesting that they were either processing information from the mouth using peripheral vision or, as MacDonald et al. (2000) suggested, that subtle movements of the jaw are sufficient to produce the McGurk effect. Perception of the McGurk effect was related to where people looked on any given trial; dwell time on the mouth tended to be greater on trials where the McGurk effect was perceived than on trials where it was not. We additionally hypothesized that effect would be driven by those who were shown a peripheral fixation cross, as has been suggested by previous research (Arizpe et al., 2012; Gurler et al., 2015). The direction of the results was in the direction predicted, but the interaction between McGurk perception and Fixation-cross position was not significant, so further research is needed to establish whether fixation-cross position is an important consideration.

Contrary to the findings of Gurler et al. (2015), however, we did not find any evidence to support the hypothesis that participants who perceived the McGurk effect more strongly would spend more time fixating the mouth. This could be because they were attending to the mouth in their peripheral vision. Pare et al. (2003) found that when participants’ gaze was directed away from the mouth, they still reported the McGurk effect, suggesting that fixating the mouth is not a *necessary* precursor to perceiving the illusion. The present experiment supports this, as we found that participants were able to look at the nose, eyes and jaw and still perceive the McGurk effect. Therefore the McGurk effect can occur without fixating the mouth, but the likelihood of perceiving the McGurk illusion is higher when a person fixates the mouth.

Additionally, visual blur decreased dwell times on the mouth. The finding of decreased dwell time on the mouth in high levels of visual blur suggests that there was less benefit of the visual information provided by the mouth. In high visual blur, we observe decreased dwell time on the mouth coupled with increased auditory responses. This suggests that in high visual blur, participants may have been focussing their attention on the auditory component of the stimulus more (or otherwise weighting the auditory signal more highly), resulting in reduced McGurk responses.

Overall, these findings establish the level of visual degradation required to inhibit McGurk responses. This is important for understanding how single senses interact when one or both modalities are degraded.

## Vocoded condition

The Clear condition aimed to clarify how different types of auditory noise influence AV integration and eye movements; this would tell us whether time spent fixating key features of the face changes depending on the type of auditory degradation experienced. Whilst the Clear condition used visual blur and white noise, other forms of auditory degradation should be considered, such as vocoding, which degrades the speech signal both spectrally (by blurring across frequency) and temporally (by removing rapid fluctuations in amplitude over time). CI users often struggle to understand speech in noise. Therefore, it is important to study vocoded speech to understand how eye-movement strategies can aid AV integration. This would elucidate which parts of the face are important in different noise contexts. Often, hearing-impaired listeners have other age-related cognitive deficits, and it is helpful to conduct initial experiments with normal hearing listeners to inform future research with hearing impaired listeners.

We aimed to replicate the results of the Clear condition with the addition that auditory stimuli were degraded using vocoded speech presented in different levels of white noise to simulate the same encoding as a CI in background noise. Previous research shows that vocoding impairs speech perception (Qin & Oxenham, 2003). Therefore, when speech is vocoded participants may look at the mouth more compared to the Clear condition when speech was Clear and presented in white noise. It is expected that people will look at the mouth more in challenging listening conditions when speech is vocoded as well as presented in white noise compared to when the only source of noise is from vocoded speech. We also expect that the results of the clear condition will be replicated and perception of the McGurk effect will increase as auditory noise increases and decrease as visual blur increases.

### Method

The same equipment and procedure were used as in the Clear condition. Participants were the same as those who completed the Clear condition; participants completed the conditions in a counterbalanced order.

The stimuli were presented with the addition that the auditory signal was vocoded as well as presented in white noise (visual blur: no blur, mid blur, high blur × auditory noise: vocoded no noise, vocoded with mid-level white noise, vocoded with high-level white noise). Stimuli were vocoded prior to the experiment in Matlab using an 8-channel vocoder. Stimuli were band-pass filtered into eight adjacent frequency bands spaced equally on an equivalent rectangular bandwidth frequency scale between 100 Hz and 8 kHz (Glasberg & Moore, 1990) using Finite Impulse Response filters. The temporal envelope of each filter output was extracted using the Hilbert transform and used to modulate a sine wave at the central frequency value of the filter. The eight sine waves were then summed. Pilot testing, as described for the Clear condition, revealed that for vocoded speech performance fell to approximately 50% correct at an SNR of -9 dB. An SNR of 0 dB fell between this and ceiling performance levels for vocoded speech, so was chosen for the Mid auditory noise condition. Visual blurring was at 40% (mid) and 60% (high).

### Results

The same six participants were excluded as in the Clear condition, giving a sample size of 31 participants.

#### Variability in McGurk effect perception across participants and stimuli

McGurk-effect perception varied across participants, ranging from 55–92% (M= 72.9%, SD = 9.7%). There was also large variability in the perception of the McGurk effect across stimuli, as Fig. 2, Panel B, shows. With Stimulus 2 the McGurk effect was perceived 92.3% of the time (SD 25.8%), while with Stimulus 1 the McGurk effect was perceived 60.5% of the time (SD 48.9%).

#### Distribution of eye movements in each AOI

Figure 3, Panels C and D, shows the distribution of eye movements within each AOI for each stimulus. As with Clear speech, the mouth received the most dwell time, followed by the nose and then the eyes. The differences in dwell time across AOIs was significant, as expected (F _5, 155_ = 27.73, p<0.001, ${\eta}_p^2$ = 0.47). There were small variations in this pattern according to which stimulus participants were viewing and whether the stimuli were congruent or incongruent, but this pattern was broadly consistent across stimuli. There was nevertheless a significant interaction between Congruence and AOI (F _5, 155_ = 3.33, p<0.01, ${\eta}_p^2$ = 0.097); slightly more time was spent fixating the mouth and less time was spent fixating the eyes when stimuli were incongruent than when stimuli were congruent (Fig. 3). Additionally, a significant interaction between AOI and Stimulus (F _15, 465_ = 5.46, p<0.001, ${\eta}_p^2$ = 0.15) was found because the pattern of fixations in each AOI varied slightly for the different stimuli. For example, more time was spent fixating the mouth of Stimulus 1 than the mouth of other stimuli.

#### Effects of auditory noise and visual blur on McGurk responses

The effects of auditory noise and visual blur on McGurk responses for Vocoded data are shown in Panel C of Fig. 4. The fixation cross interaction did not contribute significantly to the model and was removed (ΔAIC = 1.9, Δ*X*^2^ = 0.12, p = 0.72). The interaction between Auditory noise and Visual blur was not significant and was omitted from the model (ΔAIC = 2.0, Δ*X*^2^ = 0.06, p = 0.79). The variance for all random effects was zero, therefore random effects were removed from the model. The results from the final model are shown in Table 3; this shows a significant effect of visual blur, indicating that McGurk responses fell in the presence of visual blur. There was no significant effect of Auditory noise.

**Table 3:** Vocoded speech: Multi-level modelling results from the analysis of the effect of Auditory noise, Visual blur, and Fixation cross on McGurk responses

Condition	b	SE	z-value	p-value
Intercept	0.80	0.22	3.60	<0.001
Fixation cross	0.19	0.14	1.38	0.16
Auditory noise	0.12	0.07	1.80	0.07
Visual blur	-0.68	0.07	-9.11	<0.001

#### Effects of auditory noise and visual blur on dwell times on the mouth

The fixation cross interaction did not significantly improve the model and was removed (ΔAIC = 0.00, Δχ^2^ = 2.22, p = 0.13). Adding an interaction between Auditory and Visual noise did not improve the model and was omitted (ΔAIC = 2.0, Δχ^2^ = 0.007, p = 0.92). Interactions of random effects were dropped from the model due to low variance. Multi-level modelling revealed that there was more variability in mouth dwell times associated with Participants (SD = 20.41) than with Stimuli (SD = 1.92). Figure 4 (Panel D) shows the effects of auditory noise and visual blur on dwell time on the mouth, and the results are shown in Table 4. There was a significant effect of Visual blur as Dwell times on the mouth decreased in the presence of visual blur. There was no significant effect of Auditory noise.

**Table 4:** Vocoded speech: Multi-level modelling results from the analysis of the effect of Auditory noise, Visual blur and Fixation cross on McGurk responses

Condition	b	SE	t-value	p-value
Intercept	25.45	11.62	2.19	0.03
Fixation cross	4.64	7.25	0.64	0.52
Auditory noise	1.24	13.84	0.09	0.92
Visual blur	- 2.61	1.10	-2.38	<0.05

#### Association between McGurk perception and dwell time on mouth, according to fixation-cross position

The analysis just included main effects as the interaction did not significantly improve the model (ΔAIC = 2.0, Δ*X*^2^ = 0.56, p = 0.45). Figure 5 (Panel B) shows that there was a trend for people to spend more time fixating the mouth when the McGurk effect was perceived than when it was not. This was not statistically significant (b = 2.50 (SE 1.99), t = 1.25, p = 0.25). There was no significant effect of fixation cross (b = 4.52 (SE 7.43), t = 0.60, p = 0.55).

#### Correlation between McGurk perception and dwell time on mouth

There was no significant correlation between each participant’s average McGurk perception and their dwell time on the mouth (r _31_ = 0.047, p = 0.81).

### Discussion

The Vocoded Condition aimed to establish how eye movements influence AV integration when stimuli are degraded by visual blur, vocoding and white noise. Consistent with the results from the Clear condition, variability in the McGurk effect was demonstrated with the effect being perceived between 55% and 92% of the time across participants. On average, across all noise levels, the McGurk effect was perceived 72.6% of the time, which compares to the 60.8% reported in the Clear condition. Vocoded speech here appears to have led to generally greater visual influence than in the Clear condition, likely due to the poorer intelligibility of the auditory signal when speech is vocoded. McGurk perception did not fall below 50% in any condition.

Dwell time in each AOI was similar to the Clear condition as participants spent the majority of time focused on the mouth, followed by the nose. Overall, participants spent 32.0% of the time fixating the mouth region, which is slightly higher than, but comparable to, the 27.7% in the Clear condition. More time was spent fixating the mouth when stimuli were incongruent compared to when they were congruent. Consistent with the results of the Clear condition, as visual blur increased, McGurk-effect perception decreased. Additionally, less time was spent fixating the mouth if the stimuli were presented in visual noise. Unlike the Clear condition, people were not more likely to perceive the McGurk effect if they spent longer fixating the mouth, and auditory noise did not influence time spent fixating the mouth.

Overall, the vocoded condition elucidates the influence of visual information in aiding AV integration in difficult listening situations.

## General discussion

To date it has not been well understood how auditory and visual information interact under degraded conditions, and how beneficial fixating a talker’s mouth is for AV integration under these conditions is not well understood. The present experiment investigated how the relative signal strengths of modalities in multisensory task settings affect the extent of multisensory integration as well as related eye movements. AV integration was measured by perception of the McGurk effect in different levels of auditory noise and visual blur. This is relevant for people with both auditory and visual impairments and for understanding how AV integration is influenced when information from one or more modalities is degraded.

Overall, across the clear and vocoded conditions, we found that AV integration was robust; the McGurk effect, which we defined as a change in the auditory percept, averaged 60.8% in the Clear condition and 72.6% in the Vocoded condition. Only when visual information was degraded and the auditory signal was presented with no noise did the frequency of the McGurk effect fall to below 50%. According to the Principle of Inverse Effectiveness (Meredith & Stein, 1986), we would expect McGurk responses to increase as auditory noise increases, as unisensory degradation is hypothesised to improve AV integration. Our results support this hypothesis; when there was noise in the auditory signal, perception of the McGurk effect increased and people also looked more at the mouth. In the Clear condition we found that when the visual signal was not blurred McGurk responses peaked in mid auditory noise compared to no noise or high noise. As expected, adding blur to the visual signal decreased perception of the McGurk effect and also dwell times on the mouth.

A novel aspect of the current work was our manipulation of fixation-cross position. We expected that there may be a greater effect of McGurk perception in the peripheral fixation-cross condition since participants were required to make a purposeful eye movement to the AOI, rather than being able to view the area in their peripheral vision. However, the interaction between McGurk perception and Fixation-cross position was not significant, so more research is needed to establish whether fixation-cross position is an important consideration.

Contrary to previous research (Gurler et al., 2015), we did not find that stronger perceivers of the McGurk effect tended to look more at the mouth. One explanation is that strong perceivers were able to make use of the visual information from other areas of the face. Indeed, the finding that the McGurk effect remained robust even when faces and voices were subject to severe degradation suggests that viewers were still able to glean enough visual information to produce the effect. In high visual blur when the mouth was barely discernible, the McGurk effect was still perceived (in the Clear condition 20% of the time for no auditory noise, and 58% of the time for mid auditory noise). Although viewers looked at the mouth less, focussing on other areas of the face was sufficient for the McGurk effect to be perceived. Our findings provide support for previous work measuring eye movements in visual blur (Alsius et al., 2016; Wilson et al., 2016), suggesting that viewers look at the mouth more when there was a benefit of doing so, when high spatial frequency information was intact.

The findings that in the Clear condition on the one hand people are more likely to perceive the McGurk effect when they look at the mouth, but on the other that stronger perceivers of the McGurk effect were no more likely to look at the mouth might appear contradictory. However, these results arose from different analyses. For the first analysis, dwell time on the mouth was divided according to whether people perceived the McGurk effect or not. The second analysis took the average dwell time on the mouth, regardless of whether the McGurk effect was perceived, and correlated this with the percentage of time people perceived the McGurk effect. Therefore, *across individuals*, the McGurk effect was perceived more often as dwell time on the mouth increased, but it was not the case *within individuals –* those who looked more at the mouth did not perceive the McGurk effect more.

As the second-most fixated AOI was the nose, participants could have also viewed the mouth peripherally. Moreover, dynamic articulation of syllables is not just confined to the mouth and includes movements across the whole face (Vatikiotis-Bateson et al., 1998). Whilst this suggests that fixating the mouth is not always *necessary* to perceive the McGurk effect, our results show that increased McGurk responses are observed when viewers spend more time fixating the mouth. This suggests that fixating the mouth provides richer visual information that contributes to increased illusory percepts. The finding that higher levels of auditory noise led to more time fixating the mouth supports the suggestion that in challenging listening situations people look more at the most salient aspect of the face for deriving visual speech information. This is also supported by the finding that more time was spent fixating the mouth when stimuli were incongruent than when they were congruent.

Limitations of eye-movement measures should be acknowledged. During conversation viewers may look at the eyes for social cues. However, this may be more relevant for longer speech stimuli such as sentences, whereas the present study used short stimuli (~200 ms). Future research could build on the present findings by using more naturalistic speech stimuli, for example words and sentences in comparison with the McGurk effect. Previous findings (Buchan et al., 2008) also suggest that talker identity can influence gaze, as when a different talker is presented on every trial, participants focus more on the mouth compared to when the talker was consistent across trials. This may have influenced time spent fixating the mouth in the present study as although the same four talkers were presented, talker identity was randomised across trials.

A limitation of the present study is that one type of McGurk stimulus (auditory ba + visual ga) was used per talker. We conducted pilot testing to select the stimuli that were used in the current experiment, and we chose the stimuli that produced the McGurk effect to the greatest extent. This particular syllable combination was also chosen because it is the most widely used, and therefore facilitates comparisons with previous work. We acknowledge that different participants may perceive the McGurk effect to different extents based on the particular stimulus used (Basu-Mallick, Magnotti & Beauchamp, 2015). Therefore, the results may have been influenced by the choice of particular stimuli used in the current study. However, we have been able to successfully replicate several studies that used different stimuli, and our multilevel modelling analyses also allowed us to represent variability in both participants and stimuli. A further potential issue with coding McGurk responses as anything other than the auditory signal is that errors caused by fatigue or inattention could be counted as McGurk responses. However, our findings show that McGurk responses were systematically affected by our manipulations of auditory noise and visual blur, which suggests that any such errors are likely to be minimal and have little influence on our overall pattern of results.

The present study used the McGurk effect as one measure of AV integration. Our findings here may or may not necessarily generalize to wider situations in which auditory and visual stimuli are congruent or form longer speech segments. There is an underlying assumption in the literature that strong perceivers of the McGurk effect would also be more accurate at identifying congruent speech in noise than weak perceivers of the McGurk effect, because strong perceivers would be better at integrating information. However, recent research (Van Engen et al., 2017) found that when sentences and McGurk stimuli were presented in noise (multi-talker babble), sentence recognition was not predicted by susceptibility to the McGurk effect. Therefore, care should be taken when drawing conclusions directly by comparing the McGurk effect to AV integration during everyday conversation (see Alsius et al., 2017 for a review; Van Engen et al., 2017). Further research is required to examine the McGurk effect in relation to other measures of AV integration.

The findings presented here serve to resolve some of the contradictions regarding whether or not fixating the mouth is important for McGurk perception. When the visual signal is not blurred and the mouth is fixated, this increases the likelihood of the McGurk effect being perceived. Accordingly, we would expect people to receive greater benefit from visual speech information when the visual signal is not degraded and the mouth is fixated. While the McGurk effect is still perceived to some extent when the visual signal is blurred, the results suggest that if the visual signal is blurred people will receive less benefit from visual speech information, and accordingly they will disengage from looking at the mouth. The ability to integrate auditory and visual information varies across individuals and populations including older adults (Sekiyama et al., 2014) and people with hearing impairments (Tye-Murray, Spehar, Sommers & Barcroft, 2016b). Therefore, future research should continue to examine AV integration with both auditory and visual degradation with these populations as they may rely more on visual signals. It would also be interesting to carry out a further study to establish whether directing people to look at the mouth (1) leads to greater perception of the McGurk effect, and (2) enhances the amount of visual speech benefit people receive when listening to conversational speech in noise.

The findings also demonstrate how AV integration of incongruent information is influenced by degraded stimulus presentations. The McGurk effect, a visually driven illusion, was reduced when the visual signal was degraded and increased when the auditory signal was degraded. This supports the modality appropriate hypothesis, which states that the senses are weighted based on which modality is the most reliable (Ernst & Bülthoff, 2004; Witten & Knudsen, 2005). However, even when both the auditory and visual information were severely degraded the McGurk effect was still perceived. This suggests that whilst there was a decline in McGurk responses, vision remains influential even when information from both senses is unreliable.

### Conclusion

The McGurk effect is a widely cited illusion that occurs when auditory and visual information is conflicting, and is still perceived even when the visual signal is severely degraded. Fixating the mouth is not strictly necessary for AV integration, but when speech was not vocoded AV integration increased when the visual signal was clear and the mouth was fixated. This suggests the possibility that the best strategy for greater AV integration when listening in background noise may be to fixate the mouth. Future work should examine this possibility outside of the context of perception of the McGurk effect, such as when listeners are presented with conversational speech in background noise.

## Electronic supplementary material

ESM 1 (HTML 701 kb)

ESM 2 (RMD 6.97 kb)

ESM 3 (R 3.19 kb)
