# Supplementary material for: Audio-visual integration in noise: Influence of auditory and visual stimulus degradation on eye movements and perception of the McGurk effect
Source: Atten Percept Psychophys. 2020 Jun 12;82(7):3544–57. doi: 10.3758/s13414-020-02042-x (PMC7788022; doi:10.3758/s13414-020-02042-x)
Supplement: Supplementary file 1 — (HTML 701 kb) [file 13414_2020_2042_MOESM1_ESM.html]

Paper revison 4


# Paper revison 4

#### J stacey

#### 25/02/2020

```
library(readxl)
library(lme4)
```

```
## Loading required package: Matrix
```

```
library(tidyverse)
```

```
## -- Attaching packages --------------------------------------------------------------------------------- tidyverse 1.3.0 --
```

```
## v ggplot2 3.2.1     v purrr   0.3.3
## v tibble  2.1.3     v dplyr   0.8.3
## v tidyr   1.0.2     v stringr 1.4.0
## v readr   1.3.1     v forcats 0.4.0
```

```
## -- Conflicts ------------------------------------------------------------------------------------ tidyverse_conflicts() --
## x tidyr::expand() masks Matrix::expand()
## x dplyr::filter() masks stats::filter()
## x dplyr::lag()    masks stats::lag()
## x tidyr::pack()   masks Matrix::pack()
## x tidyr::unpack() masks Matrix::unpack()
```

```
library(dplyr)
library(afex)#to get p values for anova
```

```
## Registered S3 methods overwritten by 'car':
##   method                          from
##   influence.merMod                lme4
##   cooks.distance.influence.merMod lme4
##   dfbeta.influence.merMod         lme4
##   dfbetas.influence.merMod        lme4
```

```
## ************
## Welcome to afex. For support visit: http://afex.singmann.science/
```

```
## - Functions for ANOVAs: aov_car(), aov_ez(), and aov_4()
## - Methods for calculating p-values with mixed(): 'KR', 'S', 'LRT', and 'PB'
## - 'afex_aov' and 'mixed' objects can be passed to emmeans() for follow-up tests
## - NEWS: library('emmeans') now needs to be called explicitly!
## - Get and set global package options with: afex_options()
## - Set orthogonal sum-to-zero contrasts globally: set_sum_contrasts()
## - For example analyses see: browseVignettes("afex")
## ************
```

```
## 
## Attaching package: 'afex'
```

```
## The following object is masked from 'package:lme4':
## 
##     lmer
```

```
library(lmerTest)#to get p values
```

```
## 
## Attaching package: 'lmerTest'
## 
## The following object is masked from 'package:lme4':
## 
##     lmer
```

```
## The following object is masked from 'package:stats':
## 
##     step
```

```
setwd("C:/Users/mszjes/Desktop/Jammy/paper_rev_3")
Df <- read_excel("Clear_R_4.xlsx")
```

```
#mean center the fixed effect variables 
Z_Df <- Df %>%
  mutate_at(c("Stimulus_deg_V", "Stimulus_deg_A"),scale)
```

## 1. Clear speech: Effects of Auditory noise and visual blur on McGurk responses.

```
#full model with fixed effects and random effects interactions 
Model1 <- glmer(Accuracy ~ Fixation_cross + Stimulus_deg_A * Stimulus_deg_V + (1+Stimulus_deg_A*Stimulus_deg_V|Participant) + (1+ Stimulus_deg_A*Stimulus_deg_V | Stimulus_talker), data=Z_Df, family = binomial(link = "logit"))
```

```
## Warning in checkConv(attr(opt, "derivs"), opt$par, ctrl = control$checkConv, :
## Model failed to converge with max|grad| = 0.0264834 (tol = 0.001, component 1)
```

```
summary(Model1)
```

```
## Generalized linear mixed model fit by maximum likelihood (Laplace
##   Approximation) [glmerMod]
##  Family: binomial  ( logit )
## Formula: Accuracy ~ Fixation_cross + Stimulus_deg_A * Stimulus_deg_V +  
##     (1 + Stimulus_deg_A * Stimulus_deg_V | Participant) + (1 +  
##     Stimulus_deg_A * Stimulus_deg_V | Stimulus_talker)
##    Data: Z_Df
## 
##      AIC      BIC   logLik deviance df.resid 
##   1055.3   1180.7   -502.6   1005.3     1091 
## 
## Scaled residuals: 
##     Min      1Q  Median      3Q     Max 
## -6.4631 -0.4561  0.1608  0.5383  5.9528 
## 
## Random effects:
##  Groups          Name                          Variance Std.Dev. Corr       
##  Participant     (Intercept)                   0.305015 0.55228             
##                  Stimulus_deg_A                0.107264 0.32751   0.33      
##                  Stimulus_deg_V                0.006177 0.07859  -0.20  0.86
##                  Stimulus_deg_A:Stimulus_deg_V 0.015958 0.12632   0.02  0.95
##  Stimulus_talker (Intercept)                   1.969261 1.40330             
##                  Stimulus_deg_A                0.175227 0.41860   0.85      
##                  Stimulus_deg_V                0.163030 0.40377   0.74  0.98
##                  Stimulus_deg_A:Stimulus_deg_V 0.074029 0.27208  -0.43  0.11
##       
##       
##       
##       
##   0.98
##       
##       
##       
##   0.30
## Number of obs: 1116, groups:  Participant, 31; Stimulus_talker, 4
## 
## Fixed effects:
##                               Estimate Std. Error z value Pr(>|z|)    
## (Intercept)                    0.92070    0.81871   1.125    0.261    
## Fixation_cross                -0.04068    0.25541  -0.159    0.873    
## Stimulus_deg_A                 1.30161    0.24648   5.281 1.29e-07 ***
## Stimulus_deg_V                -0.93537    0.23474  -3.985 6.76e-05 ***
## Stimulus_deg_A:Stimulus_deg_V  0.07260    0.17990   0.404    0.687    
## ---
## Signif. codes:  0 '***' 0.001 '**' 0.01 '*' 0.05 '.' 0.1 ' ' 1
## 
## Correlation of Fixed Effects:
##             (Intr) Fxtn_c Stm__A Stm__V
## Fixatn_crss -0.479                     
## Stimls_dg_A  0.674 -0.019              
## Stimls_dg_V  0.511  0.023  0.641       
## Stm__A:S__V -0.325  0.030  0.048  0.360
## convergence code: 0
## Model failed to converge with max|grad| = 0.0264834 (tol = 0.001, component 1)
```

#same model without fixed effect interaction

```
Model1noInt <- glmer(Accuracy ~ Fixation_cross + Stimulus_deg_A + Stimulus_deg_V + (1+Stimulus_deg_A*Stimulus_deg_V|Participant) + (1+ Stimulus_deg_A*Stimulus_deg_V | Stimulus_talker), data=Z_Df, family = binomial(link = "logit"))
```

```
## boundary (singular) fit: see ?isSingular
```

```
summary(Model1noInt)
```

```
## Generalized linear mixed model fit by maximum likelihood (Laplace
##   Approximation) [glmerMod]
##  Family: binomial  ( logit )
## Formula: Accuracy ~ Fixation_cross + Stimulus_deg_A + Stimulus_deg_V +  
##     (1 + Stimulus_deg_A * Stimulus_deg_V | Participant) + (1 +  
##     Stimulus_deg_A * Stimulus_deg_V | Stimulus_talker)
##    Data: Z_Df
## 
##      AIC      BIC   logLik deviance df.resid 
##   1053.4   1173.8   -502.7   1005.4     1092 
## 
## Scaled residuals: 
##     Min      1Q  Median      3Q     Max 
## -6.7186 -0.4579  0.1565  0.5419  5.8530 
## 
## Random effects:
##  Groups          Name                          Variance Std.Dev. Corr       
##  Participant     (Intercept)                   0.306392 0.55353             
##                  Stimulus_deg_A                0.107065 0.32721   0.34      
##                  Stimulus_deg_V                0.006244 0.07902  -0.27  0.81
##                  Stimulus_deg_A:Stimulus_deg_V 0.015238 0.12344  -0.01  0.93
##  Stimulus_talker (Intercept)                   1.982459 1.40800             
##                  Stimulus_deg_A                0.179382 0.42353   0.85      
##                  Stimulus_deg_V                0.149361 0.38647   0.70  0.97
##                  Stimulus_deg_A:Stimulus_deg_V 0.079985 0.28282  -0.48  0.04
##       
##       
##       
##       
##   0.97
##       
##       
##       
##   0.29
## Number of obs: 1116, groups:  Participant, 31; Stimulus_talker, 4
## 
## Fixed effects:
##                Estimate Std. Error z value Pr(>|z|)    
## (Intercept)     1.03895    0.76818   1.352    0.176    
## Fixation_cross -0.04375    0.25481  -0.172    0.864    
## Stimulus_deg_A  1.30251    0.25708   5.067 4.05e-07 ***
## Stimulus_deg_V -0.96842    0.21168  -4.575 4.77e-06 ***
## ---
## Signif. codes:  0 '***' 0.001 '**' 0.01 '*' 0.05 '.' 0.1 ' ' 1
## 
## Correlation of Fixed Effects:
##             (Intr) Fxtn_c Stm__A
## Fixatn_crss -0.500              
## Stimls_dg_A  0.732 -0.020       
## Stimls_dg_V  0.696  0.013  0.658
## convergence code: 0
## boundary (singular) fit: see ?isSingular
```

#compare models to see if interaction needed

```
anova(Model1,Model1noInt)
```

```
## Data: Z_Df
## Models:
## Model1noInt: Accuracy ~ Fixation_cross + Stimulus_deg_A + Stimulus_deg_V + 
## Model1noInt:     (1 + Stimulus_deg_A * Stimulus_deg_V | Participant) + (1 + 
## Model1noInt:     Stimulus_deg_A * Stimulus_deg_V | Stimulus_talker)
## Model1: Accuracy ~ Fixation_cross + Stimulus_deg_A * Stimulus_deg_V + 
## Model1:     (1 + Stimulus_deg_A * Stimulus_deg_V | Participant) + (1 + 
## Model1:     Stimulus_deg_A * Stimulus_deg_V | Stimulus_talker)
##             Df    AIC    BIC  logLik deviance  Chisq Chi Df Pr(>Chisq)
## Model1noInt 24 1053.4 1173.8 -502.71   1005.4                         
## Model1      25 1055.3 1180.7 -502.64   1005.3 0.1534      1     0.6953
```

#interaction not needed now compare models with interaction for random effects

```
Model2noInt <- glmer(Accuracy ~ Fixation_cross + Stimulus_deg_A + Stimulus_deg_V + (1+Stimulus_deg_A+Stimulus_deg_V|Participant) + (1+ Stimulus_deg_A+Stimulus_deg_V | Stimulus_talker), data=Z_Df, family = binomial(link = "logit"))
```

```
## Warning in checkConv(attr(opt, "derivs"), opt$par, ctrl = control$checkConv, :
## Model failed to converge with max|grad| = 0.00491017 (tol = 0.001, component 1)
```

```
summary(Model2noInt)
```

```
## Generalized linear mixed model fit by maximum likelihood (Laplace
##   Approximation) [glmerMod]
##  Family: binomial  ( logit )
## Formula: Accuracy ~ Fixation_cross + Stimulus_deg_A + Stimulus_deg_V +  
##     (1 + Stimulus_deg_A + Stimulus_deg_V | Participant) + (1 +  
##     Stimulus_deg_A + Stimulus_deg_V | Stimulus_talker)
##    Data: Z_Df
## 
##      AIC      BIC   logLik deviance df.resid 
##   1048.0   1128.3   -508.0   1016.0     1100 
## 
## Scaled residuals: 
##     Min      1Q  Median      3Q     Max 
## -6.3653 -0.4973  0.1597  0.5349  3.8657 
## 
## Random effects:
##  Groups          Name           Variance Std.Dev. Corr       
##  Participant     (Intercept)    0.29508  0.54321             
##                  Stimulus_deg_A 0.10142  0.31846   0.38      
##                  Stimulus_deg_V 0.00492  0.07014  -0.67  0.44
##  Stimulus_talker (Intercept)    1.86656  1.36622             
##                  Stimulus_deg_A 0.17464  0.41790  0.92       
##                  Stimulus_deg_V 0.14917  0.38623  0.73  0.94 
## Number of obs: 1116, groups:  Participant, 31; Stimulus_talker, 4
## 
## Fixed effects:
##                Estimate Std. Error z value Pr(>|z|)    
## (Intercept)     0.94766    0.79391   1.194    0.233    
## Fixation_cross -0.03764    0.24653  -0.153    0.879    
## Stimulus_deg_A  1.26375    0.24336   5.193 2.07e-07 ***
## Stimulus_deg_V -0.90047    0.21371  -4.214 2.51e-05 ***
## ---
## Signif. codes:  0 '***' 0.001 '**' 0.01 '*' 0.05 '.' 0.1 ' ' 1
## 
## Correlation of Fixed Effects:
##             (Intr) Fxtn_c Stm__A
## Fixatn_crss -0.474              
## Stimls_dg_A  0.728 -0.005       
## Stimls_dg_V  0.556  0.004  0.689
## convergence code: 0
## Model failed to converge with max|grad| = 0.00491017 (tol = 0.001, component 1)
```

```
anova(Model2noInt,Model1noInt)
```

```
## Data: Z_Df
## Models:
## Model2noInt: Accuracy ~ Fixation_cross + Stimulus_deg_A + Stimulus_deg_V + 
## Model2noInt:     (1 + Stimulus_deg_A + Stimulus_deg_V | Participant) + (1 + 
## Model2noInt:     Stimulus_deg_A + Stimulus_deg_V | Stimulus_talker)
## Model1noInt: Accuracy ~ Fixation_cross + Stimulus_deg_A + Stimulus_deg_V + 
## Model1noInt:     (1 + Stimulus_deg_A * Stimulus_deg_V | Participant) + (1 + 
## Model1noInt:     Stimulus_deg_A * Stimulus_deg_V | Stimulus_talker)
##             Df    AIC    BIC  logLik deviance  Chisq Chi Df Pr(>Chisq)
## Model2noInt 16 1048.0 1128.3 -508.00   1016.0                         
## Model1noInt 24 1053.4 1173.8 -502.71   1005.4 10.574      8      0.227
```

#Use Model2noInt, looking at the output for this model there is low variance for visual\_deg\_V | participant and also high correlations for the talker random effect. No interactions will be specified in final model. Remove random effects with low variance and drop random effects with high correlations to prevent overfitting and solve convergence issues. #use || to remove all correlations #or (1+IV2|Subject) + (0+IV1|Subject) to specify some correlations but not others

#final model

```
Model3noInt <- glmer(Accuracy ~ Fixation_cross + Stimulus_deg_A + Stimulus_deg_V + (1+Stimulus_deg_A|Participant) + (1+ Stimulus_deg_A+Stimulus_deg_V || Stimulus_talker), data=Z_Df, family = binomial(link = "logit"))
summary(Model3noInt)
```

```
## Generalized linear mixed model fit by maximum likelihood (Laplace
##   Approximation) [glmerMod]
##  Family: binomial  ( logit )
## Formula: Accuracy ~ Fixation_cross + Stimulus_deg_A + Stimulus_deg_V +  
##     (1 + Stimulus_deg_A | Participant) + (1 + Stimulus_deg_A +  
##     Stimulus_deg_V || Stimulus_talker)
##    Data: Z_Df
## 
##      AIC      BIC   logLik deviance df.resid 
##   1046.6   1096.7   -513.3   1026.6     1106 
## 
## Scaled residuals: 
##     Min      1Q  Median      3Q     Max 
## -5.5975 -0.5010  0.1805  0.5283  3.6584 
## 
## Random effects:
##  Groups            Name           Variance Std.Dev. Corr
##  Participant       (Intercept)    0.2878   0.5364       
##                    Stimulus_deg_A 0.1069   0.3269   0.28
##  Stimulus_talker   (Intercept)    1.5904   1.2611       
##  Stimulus_talker.1 Stimulus_deg_A 0.1113   0.3336       
##  Stimulus_talker.2 Stimulus_deg_V 0.1475   0.3840       
## Number of obs: 1116, groups:  Participant, 31; Stimulus_talker, 4
## 
## Fixed effects:
##                Estimate Std. Error z value Pr(>|z|)    
## (Intercept)     0.85396    0.74781   1.142    0.253    
## Fixation_cross -0.02296    0.24730  -0.093    0.926    
## Stimulus_deg_A  1.20732    0.20510   5.886 3.95e-09 ***
## Stimulus_deg_V -0.91563    0.21165  -4.326 1.52e-05 ***
## ---
## Signif. codes:  0 '***' 0.001 '**' 0.01 '*' 0.05 '.' 0.1 ' ' 1
## 
## Correlation of Fixed Effects:
##             (Intr) Fxtn_c Stm__A
## Fixatn_crss -0.503              
## Stimls_dg_A  0.044 -0.004       
## Stimls_dg_V -0.009  0.000 -0.055
```

## 2.Clear Speech: effects of audiotry noise and visual blur on dwell times on the mouth

#full model

```
model4Int1 <- lmer(Dwell_time ~ Fixation_cross * Stimulus_deg_A+Stimulus_deg_V + (1+Stimulus_deg_A*Stimulus_deg_V|Participant) + (1+Stimulus_deg_A*Stimulus_deg_V|Stimulus_talker), data=Z_Df, REML = TRUE)
```

```
## boundary (singular) fit: see ?isSingular
```

```
summary (model4Int1)
```

```
## Linear mixed model fit by REML. t-tests use Satterthwaite's method [
## lmerModLmerTest]
## Formula: Dwell_time ~ Fixation_cross * Stimulus_deg_A + Stimulus_deg_V +  
##     (1 + Stimulus_deg_A * Stimulus_deg_V | Participant) + (1 +  
##     Stimulus_deg_A * Stimulus_deg_V | Stimulus_talker)
##    Data: Z_Df
## 
## REML criterion at convergence: 9900.8
## 
## Scaled residuals: 
##     Min      1Q  Median      3Q     Max 
## -3.6165 -0.6046 -0.0273  0.5906  3.0386 
## 
## Random effects:
##  Groups          Name                          Variance Std.Dev. Corr       
##  Participant     (Intercept)                   377.5119 19.4297             
##                  Stimulus_deg_A                  3.2951  1.8152   0.13      
##                  Stimulus_deg_V                 28.3380  5.3233   0.08  0.54
##                  Stimulus_deg_A:Stimulus_deg_V   2.0659  1.4373   0.21 -0.48
##  Stimulus_talker (Intercept)                    30.3459  5.5087             
##                  Stimulus_deg_A                  0.7659  0.8752   0.95      
##                  Stimulus_deg_V                  4.1479  2.0366   0.30  0.32
##                  Stimulus_deg_A:Stimulus_deg_V   1.8987  1.3779   0.00  0.23
##  Residual                                      361.6614 19.0174             
##       
##       
##       
##       
##  -0.96
##       
##       
##       
##  -0.55
##       
## Number of obs: 1116, groups:  Participant, 31; Stimulus_talker, 4
## 
## Fixed effects:
##                               Estimate Std. Error      df t value Pr(>|t|)  
## (Intercept)                    17.6075    11.5329 31.4743   1.527   0.1368  
## Fixation_cross                  6.4827     7.0100 28.9986   0.925   0.3627  
## Stimulus_deg_A                  0.0627     2.0903 29.4242   0.030   0.9763  
## Stimulus_deg_V                 -3.5459     1.3505  6.1574  -2.626   0.0384 *
## Fixation_cross:Stimulus_deg_A   0.4215     1.2769 29.0227   0.330   0.7437  
## ---
## Signif. codes:  0 '***' 0.001 '**' 0.01 '*' 0.05 '.' 0.1 ' ' 1
## 
## Correlation of Fixed Effects:
##             (Intr) Fxtn_c Stm__A Stm__V
## Fixatn_crss -0.922                     
## Stimls_dg_A  0.097 -0.047              
## Stimls_dg_V  0.080  0.000  0.119       
## Fxtn_c:S__A -0.047  0.051 -0.926  0.000
## convergence code: 0
## boundary (singular) fit: see ?isSingular
```

#model without main effects interaction

```
model4noInt <- lmer(Dwell_time ~ Fixation_cross + Stimulus_deg_A+Stimulus_deg_V + (1+Stimulus_deg_A*Stimulus_deg_V|Participant) + (1+Stimulus_deg_A*Stimulus_deg_V|Stimulus_talker), data=Z_Df,REML = TRUE)
```

```
## Warning in checkConv(attr(opt, "derivs"), opt$par, ctrl = control$checkConv, :
## Model failed to converge with max|grad| = 0.0240692 (tol = 0.002, component 1)
```

```
summary (model4noInt)
```

```
## Linear mixed model fit by REML. t-tests use Satterthwaite's method [
## lmerModLmerTest]
## Formula: Dwell_time ~ Fixation_cross + Stimulus_deg_A + Stimulus_deg_V +  
##     (1 + Stimulus_deg_A * Stimulus_deg_V | Participant) + (1 +  
##     Stimulus_deg_A * Stimulus_deg_V | Stimulus_talker)
##    Data: Z_Df
## 
## REML criterion at convergence: 9903.3
## 
## Scaled residuals: 
##     Min      1Q  Median      3Q     Max 
## -3.6304 -0.6120 -0.0272  0.5944  3.0288 
## 
## Random effects:
##  Groups          Name                          Variance Std.Dev. Corr       
##  Participant     (Intercept)                   377.0481 19.4177             
##                  Stimulus_deg_A                  2.8408  1.6855   0.13      
##                  Stimulus_deg_V                 28.2501  5.3151   0.08  0.55
##                  Stimulus_deg_A:Stimulus_deg_V   2.0744  1.4403   0.20 -0.51
##  Stimulus_talker (Intercept)                    30.4516  5.5183             
##                  Stimulus_deg_A                  0.7599  0.8717   0.95      
##                  Stimulus_deg_V                  4.2074  2.0512   0.30  0.33
##                  Stimulus_deg_A:Stimulus_deg_V   1.9110  1.3824   0.00  0.23
##  Residual                                      361.6746 19.0177             
##       
##       
##       
##       
##  -0.96
##       
##       
##       
##  -0.55
##       
## Number of obs: 1116, groups:  Participant, 31; Stimulus_talker, 4
## 
## Fixed effects:
##                Estimate Std. Error      df t value Pr(>|t|)  
## (Intercept)     17.8051    11.5181 31.5230   1.546   0.1321  
## Fixation_cross   6.3560     6.9991 29.0310   0.908   0.3713  
## Stimulus_deg_A   0.7017     0.7778  6.2549   0.902   0.4004  
## Stimulus_deg_V  -3.5435     1.3535  6.0447  -2.618   0.0394 *
## ---
## Signif. codes:  0 '***' 0.001 '**' 0.01 '*' 0.05 '.' 0.1 ' ' 1
## 
## Correlation of Fixed Effects:
##             (Intr) Fxtn_c Stm__A
## Fixatn_crss -0.921              
## Stimls_dg_A  0.142  0.000       
## Stimls_dg_V  0.080  0.000  0.311
## convergence code: 0
## Model failed to converge with max|grad| = 0.0240692 (tol = 0.002, component 1)
```

#compare models

```
anova(model4Int1,model4noInt)
```

```
## refitting model(s) with ML (instead of REML)
```

```
## Data: Z_Df
## Models:
## model4noInt: Dwell_time ~ Fixation_cross + Stimulus_deg_A + Stimulus_deg_V + 
## model4noInt:     (1 + Stimulus_deg_A * Stimulus_deg_V | Participant) + (1 + 
## model4noInt:     Stimulus_deg_A * Stimulus_deg_V | Stimulus_talker)
## model4Int1: Dwell_time ~ Fixation_cross * Stimulus_deg_A + Stimulus_deg_V + 
## model4Int1:     (1 + Stimulus_deg_A * Stimulus_deg_V | Participant) + (1 + 
## model4Int1:     Stimulus_deg_A * Stimulus_deg_V | Stimulus_talker)
##             Df    AIC   BIC  logLik deviance  Chisq Chi Df Pr(>Chisq)
## model4noInt 25 9967.2 10093 -4958.6   9917.2                         
## model4Int1  26 9969.0 10100 -4958.5   9917.0 0.1081      1     0.7423
```

#can drop interaction now compare models with interaction between random effects

```
model5noInt <- lmer(Dwell_time ~ Fixation_cross + Stimulus_deg_A+Stimulus_deg_V + (1+Stimulus_deg_A+Stimulus_deg_V|Participant) + (1+Stimulus_deg_A+Stimulus_deg_V|Stimulus_talker), data=Z_Df,REML = TRUE)
```

```
## Warning in checkConv(attr(opt, "derivs"), opt$par, ctrl = control$checkConv, :
## Model failed to converge with max|grad| = 0.0949394 (tol = 0.002, component 1)
```

```
summary (model5noInt)
```

```
## Linear mixed model fit by REML. t-tests use Satterthwaite's method [
## lmerModLmerTest]
## Formula: Dwell_time ~ Fixation_cross + Stimulus_deg_A + Stimulus_deg_V +  
##     (1 + Stimulus_deg_A + Stimulus_deg_V | Participant) + (1 +  
##     Stimulus_deg_A + Stimulus_deg_V | Stimulus_talker)
##    Data: Z_Df
## 
## REML criterion at convergence: 9911.4
## 
## Scaled residuals: 
##     Min      1Q  Median      3Q     Max 
## -3.8292 -0.6085 -0.0378  0.6090  2.9964 
## 
## Random effects:
##  Groups          Name           Variance Std.Dev. Corr     
##  Participant     (Intercept)    377.2250 19.4223           
##                  Stimulus_deg_A   2.7063  1.6451  0.13     
##                  Stimulus_deg_V  27.8445  5.2768  0.08 0.57
##  Stimulus_talker (Intercept)     30.2915  5.5038           
##                  Stimulus_deg_A   0.6888  0.8299  0.99     
##                  Stimulus_deg_V   3.8265  1.9561  0.31 0.40
##  Residual                       366.4501 19.1429           
## Number of obs: 1116, groups:  Participant, 31; Stimulus_talker, 4
## 
## Fixed effects:
##                Estimate Std. Error      df t value Pr(>|t|)  
## (Intercept)     19.2935    11.5902 31.3886   1.665   0.1059  
## Fixation_cross   5.5626     7.0505 28.9581   0.789   0.4365  
## Stimulus_deg_A   0.7477     0.7669  6.4761   0.975   0.3646  
## Stimulus_deg_V  -4.5544     1.4777  7.9289  -3.082   0.0152 *
## ---
## Signif. codes:  0 '***' 0.001 '**' 0.01 '*' 0.05 '.' 0.1 ' ' 1
## 
## Correlation of Fixed Effects:
##             (Intr) Fxtn_c Stm__A
## Fixatn_crss -0.922              
## Stimls_dg_A  0.143  0.000       
## Stimls_dg_V  0.064  0.000  0.285
## convergence code: 0
## Model failed to converge with max|grad| = 0.0949394 (tol = 0.002, component 1)
```

#compare

```
anova(model5noInt,model4noInt)
```

```
## refitting model(s) with ML (instead of REML)
```

```
## Data: Z_Df
## Models:
## model5noInt: Dwell_time ~ Fixation_cross + Stimulus_deg_A + Stimulus_deg_V + 
## model5noInt:     (1 + Stimulus_deg_A + Stimulus_deg_V | Participant) + (1 + 
## model5noInt:     Stimulus_deg_A + Stimulus_deg_V | Stimulus_talker)
## model4noInt: Dwell_time ~ Fixation_cross + Stimulus_deg_A + Stimulus_deg_V + 
## model4noInt:     (1 + Stimulus_deg_A * Stimulus_deg_V | Participant) + (1 + 
## model4noInt:     Stimulus_deg_A * Stimulus_deg_V | Stimulus_talker)
##             Df    AIC   BIC  logLik deviance  Chisq Chi Df Pr(>Chisq)
## model5noInt 17 9959.5 10045 -4962.7   9925.5                         
## model4noInt 25 9967.2 10093 -4958.6   9917.2 8.3134      8     0.4035
```

#use model5noINt, stimulus\_deg\_A has both low variance and high correlations, remove from model

```
model6noInt <- lmer(Dwell_time ~ Fixation_cross + Stimulus_deg_A+Stimulus_deg_V + (1+Stimulus_deg_A+Stimulus_deg_V|Participant) + (1+Stimulus_deg_V|Stimulus_talker), data=Z_Df,REML = TRUE, control = lmerControl(optimizer = "Nelder_Mead"))
```

```
## Warning in checkConv(attr(opt, "derivs"), opt$par, ctrl = control$checkConv, :
## unable to evaluate scaled gradient
```

```
## Warning in checkConv(attr(opt, "derivs"), opt$par, ctrl = control$checkConv, :
## Model failed to converge: degenerate Hessian with 1 negative eigenvalues
```

```
## Warning: Model failed to converge with 1 negative eigenvalue: -2.0e+00
```

```
summary (model6noInt)
```

```
## Linear mixed model fit by REML. t-tests use Satterthwaite's method [
## lmerModLmerTest]
## Formula: Dwell_time ~ Fixation_cross + Stimulus_deg_A + Stimulus_deg_V +  
##     (1 + Stimulus_deg_A + Stimulus_deg_V | Participant) + (1 +  
##     Stimulus_deg_V | Stimulus_talker)
##    Data: Z_Df
## Control: lmerControl(optimizer = "Nelder_Mead")
## 
## REML criterion at convergence: 9923.1
## 
## Scaled residuals: 
##     Min      1Q  Median      3Q     Max 
## -3.8809 -0.5992 -0.0366  0.6214  2.9986 
## 
## Random effects:
##  Groups          Name           Variance  Std.Dev. Corr      
##  Participant     (Intercept)    4.270e+02 20.6641            
##                  Stimulus_deg_A 4.398e-02  0.2097  0.88      
##                  Stimulus_deg_V 3.002e+01  5.4794  0.08  0.51
##  Stimulus_talker (Intercept)    1.061e+03 32.5784            
##                  Stimulus_deg_V 4.134e+00  2.0331  -0.18     
##  Residual                       3.669e+02 19.1539            
## Number of obs: 1116, groups:  Participant, 31; Stimulus_talker, 4
## 
## Fixed effects:
##                Estimate Std. Error       df t value Pr(>|t|)  
## (Intercept)     19.0870    20.2045 157.3354   0.945   0.3463  
## Fixation_cross   5.6988     7.4850  24.0244   0.761   0.4539  
## Stimulus_deg_A   0.7477     0.5748 763.2767   1.301   0.1938  
## Stimulus_deg_V  -4.5544     1.5267   2.0407  -2.983   0.0941 .
## ---
## Signif. codes:  0 '***' 0.001 '**' 0.01 '*' 0.05 '.' 0.1 ' ' 1
## 
## Correlation of Fixed Effects:
##             (Intr) Fxtn_c Stm__A
## Fixatn_crss -0.562              
## Stimls_dg_A  0.011  0.000       
## Stimls_dg_V -0.087  0.000  0.022
## convergence code: 0
## unable to evaluate scaled gradient
## Model failed to converge: degenerate  Hessian with 1 negative eigenvalues
```

#model fails to converge, removing high and low correlations from model

```
model7noInt <- lmer(Dwell_time ~ Fixation_cross + Stimulus_deg_A+Stimulus_deg_V+ (1+Stimulus_deg_A+Stimulus_deg_V||Participant) + (1+Stimulus_deg_V||Stimulus_talker), data=Z_Df, REML = TRUE, control = lmerControl(optimizer = "Nelder_Mead"))
summary (model7noInt)
```

```
## Linear mixed model fit by REML. t-tests use Satterthwaite's method [
## lmerModLmerTest]
## Formula: Dwell_time ~ Fixation_cross + Stimulus_deg_A + Stimulus_deg_V +  
##     (1 + Stimulus_deg_A + Stimulus_deg_V || Participant) + (1 +  
##     Stimulus_deg_V || Stimulus_talker)
##    Data: Z_Df
## Control: lmerControl(optimizer = "Nelder_Mead")
## 
## REML criterion at convergence: 9914.9
## 
## Scaled residuals: 
##     Min      1Q  Median      3Q     Max 
## -3.8613 -0.5815 -0.0406  0.6190  3.0061 
## 
## Random effects:
##  Groups            Name           Variance Std.Dev.
##  Participant       (Intercept)    376.844  19.412  
##  Participant.1     Stimulus_deg_A   2.675   1.636  
##  Participant.2     Stimulus_deg_V  27.822   5.275  
##  Stimulus_talker   (Intercept)     30.401   5.514  
##  Stimulus_talker.1 Stimulus_deg_V   3.813   1.953  
##  Residual                         367.064  19.159  
## Number of obs: 1116, groups:  Participant, 31; Stimulus_talker, 4
## 
## Fixed effects:
##                Estimate Std. Error      df t value Pr(>|t|)  
## (Intercept)     20.0982    11.6190 31.3881   1.730   0.0935 .
## Fixation_cross   5.0318     7.0706 29.0002   0.712   0.4824  
## Stimulus_deg_A   0.7477     0.6446 30.0002   1.160   0.2552  
## Stimulus_deg_V  -4.5544     1.4764  7.9412  -3.085   0.0151 *
## ---
## Signif. codes:  0 '***' 0.001 '**' 0.01 '*' 0.05 '.' 0.1 ' ' 1
## 
## Correlation of Fixed Effects:
##             (Intr) Fxtn_c Stm__A
## Fixatn_crss -0.923              
## Stimls_dg_A  0.000  0.000       
## Stimls_dg_V  0.000  0.000  0.000
```

#check that all models have REML = TRUE, then for the final model use the optimizer to get it to converge

#compare full model with no int with model with int #Now examine other interaction A\*V

```
model4Int2 <- lmer(Dwell_time ~ Fixation_cross + Stimulus_deg_A*Stimulus_deg_V + (1+Stimulus_deg_A*Stimulus_deg_V|Participant) + (1+Stimulus_deg_A*Stimulus_deg_V|Stimulus_talker), data=Z_Df, REML = TRUE)
```

```
## boundary (singular) fit: see ?isSingular
```

```
summary (model4Int2)
```

```
## Linear mixed model fit by REML. t-tests use Satterthwaite's method [
## lmerModLmerTest]
## Formula: Dwell_time ~ Fixation_cross + Stimulus_deg_A * Stimulus_deg_V +  
##     (1 + Stimulus_deg_A * Stimulus_deg_V | Participant) + (1 +  
##     Stimulus_deg_A * Stimulus_deg_V | Stimulus_talker)
##    Data: Z_Df
## 
## REML criterion at convergence: 9899
## 
## Scaled residuals: 
##     Min      1Q  Median      3Q     Max 
## -3.6197 -0.6095 -0.0278  0.6001  3.0249 
## 
## Random effects:
##  Groups          Name                          Variance Std.Dev. Corr       
##  Participant     (Intercept)                   376.6364 19.4071             
##                  Stimulus_deg_A                  2.8254  1.6809   0.13      
##                  Stimulus_deg_V                 28.1839  5.3088   0.08  0.55
##                  Stimulus_deg_A:Stimulus_deg_V   2.0001  1.4143   0.21 -0.51
##  Stimulus_talker (Intercept)                    30.6066  5.5323             
##                  Stimulus_deg_A                  0.7331  0.8562   0.97      
##                  Stimulus_deg_V                  3.8534  1.9630   0.31  0.36
##                  Stimulus_deg_A:Stimulus_deg_V   0.7091  0.8421   0.00  0.15
##  Residual                                      361.6877 19.0181             
##       
##       
##       
##       
##  -0.96
##       
##       
##       
##  -0.57
##       
## Number of obs: 1116, groups:  Participant, 31; Stimulus_talker, 4
## 
## Fixed effects:
##                               Estimate Std. Error      df t value Pr(>|t|)  
## (Intercept)                    18.1099    11.5166 31.5980   1.572   0.1258  
## Fixation_cross                  6.3432     6.9961 29.0753   0.907   0.3720  
## Stimulus_deg_A                  0.7477     0.7738  6.2608   0.966   0.3698  
## Stimulus_deg_V                 -4.5544     1.4822  8.0092  -3.073   0.0153 *
## Stimulus_deg_A:Stimulus_deg_V   1.3965     0.7526  4.0087   1.856   0.1370  
## ---
## Signif. codes:  0 '***' 0.001 '**' 0.01 '*' 0.05 '.' 0.1 ' ' 1
## 
## Correlation of Fixed Effects:
##             (Intr) Fxtn_c Stm__A Stm__V
## Fixatn_crss -0.921                     
## Stimls_dg_A  0.144  0.000              
## Stimls_dg_V  0.065  0.000  0.272       
## Stm__A:S__V  0.021  0.000 -0.020 -0.420
## convergence code: 0
## boundary (singular) fit: see ?isSingular
```

```
model5Int2 <- lmer(Dwell_time ~ Fixation_cross + Stimulus_deg_A+Stimulus_deg_V + (1+Stimulus_deg_A*Stimulus_deg_V|Participant) + (1+Stimulus_deg_A*Stimulus_deg_V|Stimulus_talker), data=Z_Df, REML = TRUE)
```

```
## Warning in checkConv(attr(opt, "derivs"), opt$par, ctrl = control$checkConv, :
## Model failed to converge with max|grad| = 0.0240692 (tol = 0.002, component 1)
```

```
summary (model5Int2)
```

```
## Linear mixed model fit by REML. t-tests use Satterthwaite's method [
## lmerModLmerTest]
## Formula: Dwell_time ~ Fixation_cross + Stimulus_deg_A + Stimulus_deg_V +  
##     (1 + Stimulus_deg_A * Stimulus_deg_V | Participant) + (1 +  
##     Stimulus_deg_A * Stimulus_deg_V | Stimulus_talker)
##    Data: Z_Df
## 
## REML criterion at convergence: 9903.3
## 
## Scaled residuals: 
##     Min      1Q  Median      3Q     Max 
## -3.6304 -0.6120 -0.0272  0.5944  3.0288 
## 
## Random effects:
##  Groups          Name                          Variance Std.Dev. Corr       
##  Participant     (Intercept)                   377.0481 19.4177             
##                  Stimulus_deg_A                  2.8408  1.6855   0.13      
##                  Stimulus_deg_V                 28.2501  5.3151   0.08  0.55
##                  Stimulus_deg_A:Stimulus_deg_V   2.0744  1.4403   0.20 -0.51
##  Stimulus_talker (Intercept)                    30.4516  5.5183             
##                  Stimulus_deg_A                  0.7599  0.8717   0.95      
##                  Stimulus_deg_V                  4.2074  2.0512   0.30  0.33
##                  Stimulus_deg_A:Stimulus_deg_V   1.9110  1.3824   0.00  0.23
##  Residual                                      361.6746 19.0177             
##       
##       
##       
##       
##  -0.96
##       
##       
##       
##  -0.55
##       
## Number of obs: 1116, groups:  Participant, 31; Stimulus_talker, 4
## 
## Fixed effects:
##                Estimate Std. Error      df t value Pr(>|t|)  
## (Intercept)     17.8051    11.5181 31.5230   1.546   0.1321  
## Fixation_cross   6.3560     6.9991 29.0310   0.908   0.3713  
## Stimulus_deg_A   0.7017     0.7778  6.2549   0.902   0.4004  
## Stimulus_deg_V  -3.5435     1.3535  6.0447  -2.618   0.0394 *
## ---
## Signif. codes:  0 '***' 0.001 '**' 0.01 '*' 0.05 '.' 0.1 ' ' 1
## 
## Correlation of Fixed Effects:
##             (Intr) Fxtn_c Stm__A
## Fixatn_crss -0.921              
## Stimls_dg_A  0.142  0.000       
## Stimls_dg_V  0.080  0.000  0.311
## convergence code: 0
## Model failed to converge with max|grad| = 0.0240692 (tol = 0.002, component 1)
```

```
anova(model4Int2,model5Int2)
```

```
## refitting model(s) with ML (instead of REML)
```

```
## Data: Z_Df
## Models:
## model5Int2: Dwell_time ~ Fixation_cross + Stimulus_deg_A + Stimulus_deg_V + 
## model5Int2:     (1 + Stimulus_deg_A * Stimulus_deg_V | Participant) + (1 + 
## model5Int2:     Stimulus_deg_A * Stimulus_deg_V | Stimulus_talker)
## model4Int2: Dwell_time ~ Fixation_cross + Stimulus_deg_A * Stimulus_deg_V + 
## model4Int2:     (1 + Stimulus_deg_A * Stimulus_deg_V | Participant) + (1 + 
## model4Int2:     Stimulus_deg_A * Stimulus_deg_V | Stimulus_talker)
##            Df    AIC   BIC  logLik deviance  Chisq Chi Df Pr(>Chisq)  
## model5Int2 25 9967.2 10093 -4958.6   9917.2                           
## model4Int2 26 9966.0 10096 -4957.0   9914.0 3.1064      1    0.07799 .
## ---
## Signif. codes:  0 '***' 0.001 '**' 0.01 '*' 0.05 '.' 0.1 ' ' 1
```

#drop A\*V interaction from model, report model7 no Int

## 3.Clear speech: Association between McGurk perception and Dwell time on mouth, according to fixation cross position

```
McGurkn <- lmer(Dwell_time ~ Fixation_cross*Accuracy + (1+Fixation_cross*Accuracy|Participant) + (1+Fixation_cross*Accuracy|Stimulus_talker), data=Z_Df, REML=TRUE)
```

```
## boundary (singular) fit: see ?isSingular
```

```
summary(McGurkn)
```

```
## Linear mixed model fit by REML. t-tests use Satterthwaite's method [
## lmerModLmerTest]
## Formula: Dwell_time ~ Fixation_cross * Accuracy + (1 + Fixation_cross *  
##     Accuracy | Participant) + (1 + Fixation_cross * Accuracy |  
##     Stimulus_talker)
##    Data: Z_Df
## 
## REML criterion at convergence: 9978.2
## 
## Scaled residuals: 
##     Min      1Q  Median      3Q     Max 
## -3.4625 -0.6674 -0.0497  0.6424  3.4942 
## 
## Random effects:
##  Groups          Name                    Variance Std.Dev. Corr             
##  Participant     (Intercept)             559.41   23.652                    
##                  Fixation_cross          105.53   10.273   -0.64            
##                  Accuracy                139.92   11.829    0.24 -0.14      
##                  Fixation_cross:Accuracy  59.02    7.682   -0.24  0.20 -1.00
##  Stimulus_talker (Intercept)              66.91    8.180                    
##                  Fixation_cross           12.70    3.564   -0.69            
##                  Accuracy                108.61   10.421   -1.00  0.75      
##                  Fixation_cross:Accuracy  61.08    7.816    0.98 -0.83 -0.99
##  Residual                                405.42   20.135                    
## Number of obs: 1116, groups:  Participant, 31; Stimulus_talker, 4
## 
## Fixed effects:
##                         Estimate Std. Error      df t value Pr(>|t|)
## (Intercept)              19.5194    11.9878 20.2950   1.628    0.119
## Fixation_cross            3.1590     7.3030 28.0018   0.433    0.669
## Accuracy                 -0.9703     7.0201  4.0203  -0.138    0.897
## Fixation_cross:Accuracy   4.6110     4.8848  3.5302   0.944    0.405
## 
## Correlation of Fixed Effects:
##             (Intr) Fxtn_c Accrcy
## Fixatn_crss -0.923              
## Accuracy    -0.298  0.187       
## Fxtn_crss:A  0.312 -0.220 -0.969
## convergence code: 0
## boundary (singular) fit: see ?isSingular
```

```
McGurknInt <- lmer(Dwell_time ~ Fixation_cross+Accuracy + (1+Fixation_cross*Accuracy|Participant) + (1+Fixation_cross*Accuracy|Stimulus_talker), data=Z_Df, REML=TRUE)
```

```
## boundary (singular) fit: see ?isSingular
```

```
summary(McGurknInt)
```

```
## Linear mixed model fit by REML. t-tests use Satterthwaite's method [
## lmerModLmerTest]
## Formula: Dwell_time ~ Fixation_cross + Accuracy + (1 + Fixation_cross *  
##     Accuracy | Participant) + (1 + Fixation_cross * Accuracy |  
##     Stimulus_talker)
##    Data: Z_Df
## 
## REML criterion at convergence: 9984.1
## 
## Scaled residuals: 
##     Min      1Q  Median      3Q     Max 
## -3.4710 -0.6789 -0.0553  0.6409  3.4833 
## 
## Random effects:
##  Groups          Name                    Variance Std.Dev. Corr             
##  Participant     (Intercept)             364.621  19.095                    
##                  Fixation_cross            8.721   2.953   -0.18            
##                  Accuracy                116.410  10.789    0.22 -0.17      
##                  Fixation_cross:Accuracy  48.153   6.939   -0.17  0.23 -0.99
##  Stimulus_talker (Intercept)              72.190   8.496                    
##                  Fixation_cross           14.365   3.790   -0.73            
##                  Accuracy                110.613  10.517   -1.00  0.78      
##                  Fixation_cross:Accuracy  62.355   7.897    0.98 -0.85 -0.99
##  Residual                                405.451  20.136                    
## Number of obs: 1116, groups:  Participant, 31; Stimulus_talker, 4
## 
## Fixed effects:
##                Estimate Std. Error     df t value Pr(>|t|)  
## (Intercept)      15.854     11.396 20.894   1.391   0.1788  
## Fixation_cross    4.812      7.122 29.589   0.676   0.5045  
## Accuracy          5.454      1.729  8.759   3.154   0.0121 *
## ---
## Signif. codes:  0 '***' 0.001 '**' 0.01 '*' 0.05 '.' 0.1 ' ' 1
## 
## Correlation of Fixed Effects:
##             (Intr) Fxtn_c
## Fixatn_crss -0.922       
## Accuracy     0.019 -0.111
## convergence code: 0
## boundary (singular) fit: see ?isSingular
```

#compare models

```
anova(McGurkn,McGurknInt)
```

```
## refitting model(s) with ML (instead of REML)
```

```
## Data: Z_Df
## Models:
## McGurknInt: Dwell_time ~ Fixation_cross + Accuracy + (1 + Fixation_cross * 
## McGurknInt:     Accuracy | Participant) + (1 + Fixation_cross * Accuracy | 
## McGurknInt:     Stimulus_talker)
## McGurkn: Dwell_time ~ Fixation_cross * Accuracy + (1 + Fixation_cross * 
## McGurkn:     Accuracy | Participant) + (1 + Fixation_cross * Accuracy | 
## McGurkn:     Stimulus_talker)
##            Df   AIC   BIC  logLik deviance  Chisq Chi Df Pr(>Chisq)
## McGurknInt 24 10045 10166 -4998.7   9997.4                         
## McGurkn    25 10046 10172 -4998.2   9996.4 1.0049      1     0.3161
```

#can drop fixed effect interaction but now overfitting, drop random effects interactions

```
McGurknInt2 <- lmer(Dwell_time ~ Fixation_cross+Accuracy + (1+Accuracy|Participant) + (1+Accuracy|Stimulus_talker), data=Z_Df, REML=TRUE)
```

```
## Warning in checkConv(attr(opt, "derivs"), opt$par, ctrl = control$checkConv, :
## Model failed to converge with max|grad| = 0.00942953 (tol = 0.002, component 1)
```

```
summary(McGurknInt2)
```

```
## Linear mixed model fit by REML. t-tests use Satterthwaite's method [
## lmerModLmerTest]
## Formula: 
## Dwell_time ~ Fixation_cross + Accuracy + (1 + Accuracy | Participant) +  
##     (1 + Accuracy | Stimulus_talker)
##    Data: Z_Df
## 
## REML criterion at convergence: 9993.5
## 
## Scaled residuals: 
##     Min      1Q  Median      3Q     Max 
## -3.4443 -0.6567 -0.0578  0.6337  3.3013 
## 
## Random effects:
##  Groups          Name        Variance  Std.Dev. Corr
##  Participant     (Intercept) 352.27406 18.7690      
##                  Accuracy     17.63418  4.1993  0.19
##  Stimulus_talker (Intercept)  35.26102  5.9381      
##                  Accuracy      0.03468  0.1862  0.97
##  Residual                    410.18539 20.2530      
## Number of obs: 1116, groups:  Participant, 31; Stimulus_talker, 4
## 
## Fixed effects:
##                Estimate Std. Error     df t value Pr(>|t|)    
## (Intercept)      18.985     11.561 31.529   1.642  0.11050    
## Fixation_cross    3.391      6.993 29.015   0.485  0.63133    
## Accuracy          6.058      1.555 32.455   3.896  0.00046 ***
## ---
## Signif. codes:  0 '***' 0.001 '**' 0.01 '*' 0.05 '.' 0.1 ' ' 1
## 
## Correlation of Fixed Effects:
##             (Intr) Fxtn_c
## Fixatn_crss -0.917       
## Accuracy    -0.022  0.001
## convergence code: 0
## Model failed to converge with max|grad| = 0.00942953 (tol = 0.002, component 1)
```

#Accuracy\_Stimulus\_talker has low variance, drop from model

```
McGurknInt2 <- lmer(Dwell_time ~ Fixation_cross+Accuracy + (1+Accuracy|Participant) + (1|Stimulus_talker), data=Z_Df, REML=TRUE)
```

```
## Warning in checkConv(attr(opt, "derivs"), opt$par, ctrl = control$checkConv, :
## Model failed to converge with max|grad| = 0.00360627 (tol = 0.002, component 1)
```

```
summary(McGurknInt2)
```

```
## Linear mixed model fit by REML. t-tests use Satterthwaite's method [
## lmerModLmerTest]
## Formula: 
## Dwell_time ~ Fixation_cross + Accuracy + (1 + Accuracy | Participant) +  
##     (1 | Stimulus_talker)
##    Data: Z_Df
## 
## REML criterion at convergence: 9993.5
## 
## Scaled residuals: 
##     Min      1Q  Median      3Q     Max 
## -3.4455 -0.6584 -0.0598  0.6310  3.3036 
## 
## Random effects:
##  Groups          Name        Variance Std.Dev. Corr
##  Participant     (Intercept) 351.84   18.757       
##                  Accuracy     17.63    4.199   0.18
##  Stimulus_talker (Intercept)  36.54    6.045       
##  Residual                    410.21   20.254       
## Number of obs: 1116, groups:  Participant, 31; Stimulus_talker, 4
## 
## Fixed effects:
##                Estimate Std. Error     df t value Pr(>|t|)    
## (Intercept)      18.951     11.569 31.799   1.638 0.111249    
## Fixation_cross    3.399      6.989 29.069   0.486 0.630345    
## Accuracy          6.073      1.553 35.656   3.910 0.000396 ***
## ---
## Signif. codes:  0 '***' 0.001 '**' 0.01 '*' 0.05 '.' 0.1 ' ' 1
## 
## Correlation of Fixed Effects:
##             (Intr) Fxtn_c
## Fixatn_crss -0.916       
## Accuracy    -0.037  0.001
## convergence code: 0
## Model failed to converge with max|grad| = 0.00360627 (tol = 0.002, component 1)
```

#overfitting, Accuracy\_Participant has low variance, remove

```
McGurknInt3 <- lmer(Dwell_time ~ Fixation_cross+Accuracy + (1|Participant) + (1|Stimulus_talker), data=Z_Df, REML=TRUE)
summary(McGurknInt3)
```

```
## Linear mixed model fit by REML. t-tests use Satterthwaite's method [
## lmerModLmerTest]
## Formula: Dwell_time ~ Fixation_cross + Accuracy + (1 | Participant) +  
##     (1 | Stimulus_talker)
##    Data: Z_Df
## 
## REML criterion at convergence: 9995.7
## 
## Scaled residuals: 
##     Min      1Q  Median      3Q     Max 
## -3.3532 -0.6515 -0.0559  0.6324  3.3665 
## 
## Random effects:
##  Groups          Name        Variance Std.Dev.
##  Participant     (Intercept) 376.58   19.406  
##  Stimulus_talker (Intercept)  36.07    6.006  
##  Residual                    414.21   20.352  
## Number of obs: 1116, groups:  Participant, 31; Stimulus_talker, 4
## 
## Fixed effects:
##                Estimate Std. Error       df t value Pr(>|t|)    
## (Intercept)      16.372     11.724   31.902   1.396    0.172    
## Fixation_cross    5.042      7.080   29.000   0.712    0.482    
## Accuracy          6.100      1.363 1082.537   4.474 8.47e-06 ***
## ---
## Signif. codes:  0 '***' 0.001 '**' 0.01 '*' 0.05 '.' 0.1 ' ' 1
## 
## Correlation of Fixed Effects:
##             (Intr) Fxtn_c
## Fixatn_crss -0.916       
## Accuracy    -0.071  0.000
```

#report model McgurknInt3

## Vocoded condition analyses

```
DfV <- read_excel("Voc_R_P.xlsx")
```

```
#mean center the fixed effect variables 
Z_DfV <- DfV %>%
  mutate_at(c("Stimulus_deg_V", "Stimulus_deg_A"),scale)
```

## 1.Vocoded speech: Effects of auditory noise and visual blur on McGUrk responses

```
Model1V <- glmer(Accuracy ~ Fixation_cross * Stimulus_deg_A + Stimulus_deg_V + (1+Stimulus_deg_A*Stimulus_deg_V|Participant) + (1+ Stimulus_deg_A*Stimulus_deg_V | Stimulus_talker), data=Z_DfV, family = binomial(link = "logit"))
```

```
## boundary (singular) fit: see ?isSingular
```

```
summary(Model1V)
```

```
## Generalized linear mixed model fit by maximum likelihood (Laplace
##   Approximation) [glmerMod]
##  Family: binomial  ( logit )
## Formula: Accuracy ~ Fixation_cross * Stimulus_deg_A + Stimulus_deg_V +  
##     (1 + Stimulus_deg_A * Stimulus_deg_V | Participant) + (1 +  
##     Stimulus_deg_A * Stimulus_deg_V | Stimulus_talker)
##    Data: Z_DfV
## 
##      AIC      BIC   logLik deviance df.resid 
##   1124.8   1250.3   -537.4   1074.8     1091 
## 
## Scaled residuals: 
##     Min      1Q  Median      3Q     Max 
## -3.8604 -0.5454  0.3083  0.4965  2.0878 
## 
## Random effects:
##  Groups          Name                          Variance Std.Dev. Corr       
##  Participant     (Intercept)                   0.204453 0.45216             
##                  Stimulus_deg_A                0.005783 0.07604  -0.66      
##                  Stimulus_deg_V                0.102390 0.31998   0.43 -0.96
##                  Stimulus_deg_A:Stimulus_deg_V 0.071190 0.26681  -0.25 -0.56
##  Stimulus_talker (Intercept)                   0.725542 0.85179             
##                  Stimulus_deg_A                0.072157 0.26862  -0.31      
##                  Stimulus_deg_V                0.168287 0.41023   1.00 -0.23
##                  Stimulus_deg_A:Stimulus_deg_V 0.001688 0.04108   0.88 -0.73
##       
##       
##       
##       
##   0.77
##       
##       
##       
##   0.83
## Number of obs: 1116, groups:  Participant, 31; Stimulus_talker, 4
## 
## Fixed effects:
##                               Estimate Std. Error z value Pr(>|z|)   
## (Intercept)                    0.86431    0.57857   1.494  0.13521   
## Fixation_cross                 0.29011    0.24134   1.202  0.22933   
## Stimulus_deg_A                 0.23752    0.29370   0.809  0.41869   
## Stimulus_deg_V                -0.68803    0.22743  -3.025  0.00248 **
## Fixation_cross:Stimulus_deg_A -0.05851    0.16522  -0.354  0.72326   
## ---
## Signif. codes:  0 '***' 0.001 '**' 0.01 '*' 0.05 '.' 0.1 ' ' 1
## 
## Correlation of Fixed Effects:
##             (Intr) Fxtn_c Stm__A Stm__V
## Fixatn_crss -0.641                     
## Stimls_dg_A -0.116  0.036              
## Stimls_dg_V  0.659 -0.007 -0.096       
## Fxtn_c:S__A  0.027 -0.048 -0.843  0.010
## convergence code: 0
## boundary (singular) fit: see ?isSingular
```

#with no interaction

```
Model2V <- glmer(Accuracy ~ Fixation_cross + Stimulus_deg_A + Stimulus_deg_V + (1+Stimulus_deg_A*Stimulus_deg_V|Participant) + (1+ Stimulus_deg_A*Stimulus_deg_V | Stimulus_talker), data=Z_DfV, family = binomial(link = "logit"))
```

```
## Warning in checkConv(attr(opt, "derivs"), opt$par, ctrl = control$checkConv, :
## Model failed to converge with max|grad| = 0.00882498 (tol = 0.001, component 1)
```

```
summary(Model2V)
```

```
## Generalized linear mixed model fit by maximum likelihood (Laplace
##   Approximation) [glmerMod]
##  Family: binomial  ( logit )
## Formula: Accuracy ~ Fixation_cross + Stimulus_deg_A + Stimulus_deg_V +  
##     (1 + Stimulus_deg_A * Stimulus_deg_V | Participant) + (1 +  
##     Stimulus_deg_A * Stimulus_deg_V | Stimulus_talker)
##    Data: Z_DfV
## 
##      AIC      BIC   logLik deviance df.resid 
##   1122.9   1243.4   -537.5   1074.9     1092 
## 
## Scaled residuals: 
##     Min      1Q  Median      3Q     Max 
## -3.8516 -0.5464  0.3069  0.4984  2.0880 
## 
## Random effects:
##  Groups          Name                          Variance Std.Dev. Corr       
##  Participant     (Intercept)                   0.204032 0.45170             
##                  Stimulus_deg_A                0.004511 0.06716  -0.73      
##                  Stimulus_deg_V                0.101923 0.31925   0.43 -0.93
##                  Stimulus_deg_A:Stimulus_deg_V 0.072019 0.26836  -0.25 -0.48
##  Stimulus_talker (Intercept)                   0.724722 0.85131             
##                  Stimulus_deg_A                0.071883 0.26811  -0.31      
##                  Stimulus_deg_V                0.168240 0.41017   1.00 -0.22
##                  Stimulus_deg_A:Stimulus_deg_V 0.001761 0.04197   0.89 -0.71
##       
##       
##       
##       
##   0.77
##       
##       
##       
##   0.84
## Number of obs: 1116, groups:  Participant, 31; Stimulus_talker, 4
## 
## Fixed effects:
##                Estimate Std. Error z value Pr(>|z|)   
## (Intercept)      0.8693     0.5781   1.504  0.13269   
## Fixation_cross   0.2868     0.2415   1.187  0.23506   
## Stimulus_deg_A   0.1500     0.1579   0.950  0.34216   
## Stimulus_deg_V  -0.6872     0.2269  -3.028  0.00246 **
## ---
## Signif. codes:  0 '***' 0.001 '**' 0.01 '*' 0.05 '.' 0.1 ' ' 1
## 
## Correlation of Fixed Effects:
##             (Intr) Fxtn_c Stm__A
## Fixatn_crss -0.642              
## Stimls_dg_A -0.175  0.001       
## Stimls_dg_V  0.657 -0.006 -0.156
## convergence code: 0
## Model failed to converge with max|grad| = 0.00882498 (tol = 0.001, component 1)
```

#compare models

```
anova(Model1V,Model2V)
```

```
## Data: Z_DfV
## Models:
## Model2V: Accuracy ~ Fixation_cross + Stimulus_deg_A + Stimulus_deg_V + 
## Model2V:     (1 + Stimulus_deg_A * Stimulus_deg_V | Participant) + (1 + 
## Model2V:     Stimulus_deg_A * Stimulus_deg_V | Stimulus_talker)
## Model1V: Accuracy ~ Fixation_cross * Stimulus_deg_A + Stimulus_deg_V + 
## Model1V:     (1 + Stimulus_deg_A * Stimulus_deg_V | Participant) + (1 + 
## Model1V:     Stimulus_deg_A * Stimulus_deg_V | Stimulus_talker)
##         Df    AIC    BIC  logLik deviance  Chisq Chi Df Pr(>Chisq)
## Model2V 24 1122.9 1243.4 -537.47   1074.9                         
## Model1V 25 1124.8 1250.3 -537.41   1074.8 0.1227      1     0.7261
```

#remove interaction, random effects not contributing to the model, remove all

```
Model3V <- glm(Accuracy ~ Fixation_cross + Stimulus_deg_A + Stimulus_deg_V, data=Z_DfV, family = binomial(link = "logit"))
summary(Model3V)
```

```
## 
## Call:
## glm(formula = Accuracy ~ Fixation_cross + Stimulus_deg_A + Stimulus_deg_V, 
##     family = binomial(link = "logit"), data = Z_DfV)
## 
## Deviance Residuals: 
##     Min       1Q   Median       3Q      Max  
## -2.1409  -1.1821   0.5446   0.7915   1.1728  
## 
## Coefficients:
##                Estimate Std. Error z value Pr(>|z|)    
## (Intercept)     0.80447    0.22311   3.606 0.000311 ***
## Fixation_cross  0.19574    0.14092   1.389 0.164810    
## Stimulus_deg_A  0.12756    0.07061   1.807 0.070840 .  
## Stimulus_deg_V -0.68054    0.07468  -9.112  < 2e-16 ***
## ---
## Signif. codes:  0 '***' 0.001 '**' 0.01 '*' 0.05 '.' 0.1 ' ' 1
## 
## (Dispersion parameter for binomial family taken to be 1)
## 
##     Null deviance: 1303.2  on 1115  degrees of freedom
## Residual deviance: 1206.6  on 1112  degrees of freedom
## AIC: 1214.6
## 
## Number of Fisher Scoring iterations: 4
```

#now check if other interaction needed

```
Model4V <- glmer(Accuracy ~ Fixation_cross + Stimulus_deg_A * Stimulus_deg_V + (1+Stimulus_deg_A*Stimulus_deg_V|Participant) + (1+ Stimulus_deg_A*Stimulus_deg_V | Stimulus_talker), data=Z_DfV, family = binomial(link = "logit"))
```

```
## Warning in checkConv(attr(opt, "derivs"), opt$par, ctrl = control$checkConv, :
## Model failed to converge with max|grad| = 0.0238802 (tol = 0.001, component 1)
```

```
summary(Model4V)
```

```
## Generalized linear mixed model fit by maximum likelihood (Laplace
##   Approximation) [glmerMod]
##  Family: binomial  ( logit )
## Formula: Accuracy ~ Fixation_cross + Stimulus_deg_A * Stimulus_deg_V +  
##     (1 + Stimulus_deg_A * Stimulus_deg_V | Participant) + (1 +  
##     Stimulus_deg_A * Stimulus_deg_V | Stimulus_talker)
##    Data: Z_DfV
## 
##      AIC      BIC   logLik deviance df.resid 
##   1124.9   1250.3   -537.4   1074.9     1091 
## 
## Scaled residuals: 
##     Min      1Q  Median      3Q     Max 
## -3.8471 -0.5474  0.3088  0.4972  2.0887 
## 
## Random effects:
##  Groups          Name                          Variance Std.Dev. Corr       
##  Participant     (Intercept)                   0.202570 0.45008             
##                  Stimulus_deg_A                0.004359 0.06603  -0.78      
##                  Stimulus_deg_V                0.099925 0.31611   0.45 -0.91
##                  Stimulus_deg_A:Stimulus_deg_V 0.069239 0.26313  -0.22 -0.43
##  Stimulus_talker (Intercept)                   0.724069 0.85092             
##                  Stimulus_deg_A                0.071488 0.26737  -0.31      
##                  Stimulus_deg_V                0.166347 0.40786   1.00 -0.22
##                  Stimulus_deg_A:Stimulus_deg_V 0.002461 0.04961   0.91 -0.67
##       
##       
##       
##       
##   0.77
##       
##       
##       
##   0.87
## Number of obs: 1116, groups:  Participant, 31; Stimulus_talker, 4
## 
## Fixed effects:
##                               Estimate Std. Error z value Pr(>|z|)   
## (Intercept)                    0.88040    0.57365   1.535  0.12485   
## Fixation_cross                 0.28985    0.24147   1.200  0.23000   
## Stimulus_deg_A                 0.13927    0.16191   0.860  0.38971   
## Stimulus_deg_V                -0.67050    0.23092  -2.904  0.00369 **
## Stimulus_deg_A:Stimulus_deg_V  0.02871    0.10577   0.271  0.78606   
## ---
## Signif. codes:  0 '***' 0.001 '**' 0.01 '*' 0.05 '.' 0.1 ' ' 1
## 
## Correlation of Fixed Effects:
##             (Intr) Fxtn_c Stm__A Stm__V
## Fixatn_crss -0.636                     
## Stimls_dg_A -0.184 -0.011              
## Stimls_dg_V  0.646  0.011 -0.186       
## Stm__A:S__V  0.107  0.048 -0.256  0.289
## convergence code: 0
## Model failed to converge with max|grad| = 0.0238802 (tol = 0.001, component 1)
```

#compare models

```
anova(Model4V,Model2V)
```

```
## Data: Z_DfV
## Models:
## Model2V: Accuracy ~ Fixation_cross + Stimulus_deg_A + Stimulus_deg_V + 
## Model2V:     (1 + Stimulus_deg_A * Stimulus_deg_V | Participant) + (1 + 
## Model2V:     Stimulus_deg_A * Stimulus_deg_V | Stimulus_talker)
## Model4V: Accuracy ~ Fixation_cross + Stimulus_deg_A * Stimulus_deg_V + 
## Model4V:     (1 + Stimulus_deg_A * Stimulus_deg_V | Participant) + (1 + 
## Model4V:     Stimulus_deg_A * Stimulus_deg_V | Stimulus_talker)
##         Df    AIC    BIC  logLik deviance  Chisq Chi Df Pr(>Chisq)
## Model2V 24 1122.9 1243.4 -537.47   1074.9                         
## Model4V 25 1124.9 1250.3 -537.44   1074.9 0.0661      1     0.7971
```

#remove interaction, report model3V

## 2. Vocoded speech: Effects of auditory noise and visual blur on Dwell times on the mouth

```
Model5V<- lmer(Dwell_time ~ Fixation_cross * Stimulus_deg_A+Stimulus_deg_V + (1+Stimulus_deg_A*Stimulus_deg_V|Stimulus_talker)+(1+Stimulus_deg_A*Stimulus_deg_V|Participant), data=Z_DfV, REML = TRUE)
```

```
## boundary (singular) fit: see ?isSingular
```

```
summary(Model5V)
```

```
## Linear mixed model fit by REML. t-tests use Satterthwaite's method [
## lmerModLmerTest]
## Formula: Dwell_time ~ Fixation_cross * Stimulus_deg_A + Stimulus_deg_V +  
##     (1 + Stimulus_deg_A * Stimulus_deg_V | Stimulus_talker) +  
##     (1 + Stimulus_deg_A * Stimulus_deg_V | Participant)
##    Data: Z_DfV
## 
## REML criterion at convergence: 9955.9
## 
## Scaled residuals: 
##     Min      1Q  Median      3Q     Max 
## -3.4223 -0.5610 -0.0510  0.6014  3.4967 
## 
## Random effects:
##  Groups          Name                          Variance Std.Dev. Corr       
##  Participant     (Intercept)                   416.4805 20.4079             
##                  Stimulus_deg_A                  1.8182  1.3484   0.79      
##                  Stimulus_deg_V                 16.9338  4.1151   0.15 -0.48
##                  Stimulus_deg_A:Stimulus_deg_V   1.0208  1.0103   0.54 -0.08
##  Stimulus_talker (Intercept)                     3.9494  1.9873             
##                  Stimulus_deg_A                  2.0853  1.4440  -0.94      
##                  Stimulus_deg_V                  1.3561  1.1645   0.37 -0.66
##                  Stimulus_deg_A:Stimulus_deg_V   0.1753  0.4187  -1.00  0.95
##  Residual                                      389.6946 19.7407             
##       
##       
##       
##       
##   0.91
##       
##       
##       
##  -0.38
##       
## Number of obs: 1116, groups:  Participant, 31; Stimulus_talker, 4
## 
## Fixed effects:
##                               Estimate Std. Error      df t value Pr(>|t|)  
## (Intercept)                     25.628     11.727  29.568   2.186   0.0369 *
## Fixation_cross                   4.522      7.309  29.022   0.619   0.5409  
## Stimulus_deg_A                   4.085      2.136  62.816   1.912   0.0604 .
## Stimulus_deg_V                  -2.627      1.105   8.088  -2.378   0.0444 *
## Fixation_cross:Stimulus_deg_A   -1.884      1.265 114.366  -1.489   0.1393  
## ---
## Signif. codes:  0 '***' 0.001 '**' 0.01 '*' 0.05 '.' 0.1 ' ' 1
## 
## Correlation of Fixed Effects:
##             (Intr) Fxtn_c Stm__A Stm__V
## Fixatn_crss -0.945                     
## Stimls_dg_A  0.270 -0.287              
## Stimls_dg_V  0.046  0.000 -0.164       
## Fxtn_c:S__A -0.302  0.320 -0.898  0.000
## convergence code: 0
## boundary (singular) fit: see ?isSingular
```

#now model with no interaction

```
Model6V<- lmer(Dwell_time ~ Fixation_cross + Stimulus_deg_A+Stimulus_deg_V + (1+Stimulus_deg_A*Stimulus_deg_V|Stimulus_talker)+(1+Stimulus_deg_A*Stimulus_deg_V|Participant), data=Z_DfV, REML = TRUE)
```

```
## boundary (singular) fit: see ?isSingular
```

```
summary(Model6V)
```

```
## Linear mixed model fit by REML. t-tests use Satterthwaite's method [
## lmerModLmerTest]
## Formula: Dwell_time ~ Fixation_cross + Stimulus_deg_A + Stimulus_deg_V +  
##     (1 + Stimulus_deg_A * Stimulus_deg_V | Stimulus_talker) +  
##     (1 + Stimulus_deg_A * Stimulus_deg_V | Participant)
##    Data: Z_DfV
## 
## REML criterion at convergence: 9960.4
## 
## Scaled residuals: 
##     Min      1Q  Median      3Q     Max 
## -3.4166 -0.5642 -0.0403  0.5948  3.5421 
## 
## Random effects:
##  Groups          Name                          Variance Std.Dev. Corr       
##  Participant     (Intercept)                   418.4188 20.455              
##                  Stimulus_deg_A                  1.9129  1.383    0.81      
##                  Stimulus_deg_V                 16.9159  4.113    0.16 -0.46
##                  Stimulus_deg_A:Stimulus_deg_V   1.0185  1.009    0.54 -0.07
##  Stimulus_talker (Intercept)                     3.9470  1.987              
##                  Stimulus_deg_A                  2.0841  1.444   -0.94      
##                  Stimulus_deg_V                  1.3576  1.165    0.37 -0.66
##                  Stimulus_deg_A:Stimulus_deg_V   0.1755  0.419   -1.00  0.95
##  Residual                                      390.0959 19.751              
##       
##       
##       
##       
##   0.92
##       
##       
##       
##  -0.38
##       
## Number of obs: 1116, groups:  Participant, 31; Stimulus_talker, 4
## 
## Fixed effects:
##                Estimate Std. Error      df t value Pr(>|t|)  
## (Intercept)     20.1831    11.1663 30.7174   1.808   0.0805 .
## Fixation_cross   8.1139     6.9147 29.5829   1.173   0.2500  
## Stimulus_deg_A   1.2296     0.9417  4.2224   1.306   0.2582  
## Stimulus_deg_V  -2.6274     1.1050  8.0672  -2.378   0.0445 *
## ---
## Signif. codes:  0 '***' 0.001 '**' 0.01 '*' 0.05 '.' 0.1 ' ' 1
## 
## Correlation of Fixed Effects:
##             (Intr) Fxtn_c Stm__A
## Fixatn_crss -0.939              
## Stimls_dg_A  0.001  0.000       
## Stimls_dg_V  0.049  0.000 -0.371
## convergence code: 0
## boundary (singular) fit: see ?isSingular
```

#compare models to see if interaction needed

```
anova(Model5V,Model6V)
```

```
## refitting model(s) with ML (instead of REML)
```

```
## Data: Z_DfV
## Models:
## Model6V: Dwell_time ~ Fixation_cross + Stimulus_deg_A + Stimulus_deg_V + 
## Model6V:     (1 + Stimulus_deg_A * Stimulus_deg_V | Stimulus_talker) + 
## Model6V:     (1 + Stimulus_deg_A * Stimulus_deg_V | Participant)
## Model5V: Dwell_time ~ Fixation_cross * Stimulus_deg_A + Stimulus_deg_V + 
## Model5V:     (1 + Stimulus_deg_A * Stimulus_deg_V | Stimulus_talker) + 
## Model5V:     (1 + Stimulus_deg_A * Stimulus_deg_V | Participant)
##         Df   AIC   BIC  logLik deviance  Chisq Chi Df Pr(>Chisq)
## Model6V 25 10024 10149 -4987.0   9974.0                         
## Model5V 26 10024 10154 -4985.9   9971.8 2.2206      1     0.1362
```

#can drop interaction, now model other interaction

```
Model7V<- lmer(Dwell_time ~ Fixation_cross + Stimulus_deg_A*Stimulus_deg_V + (1+Stimulus_deg_A*Stimulus_deg_V|Stimulus_talker)+(1+Stimulus_deg_A*Stimulus_deg_V|Participant), data=Z_DfV, REML = TRUE)
```

```
## boundary (singular) fit: see ?isSingular
```

```
summary(Model7V)
```

```
## Linear mixed model fit by REML. t-tests use Satterthwaite's method [
## lmerModLmerTest]
## Formula: Dwell_time ~ Fixation_cross + Stimulus_deg_A * Stimulus_deg_V +  
##     (1 + Stimulus_deg_A * Stimulus_deg_V | Stimulus_talker) +  
##     (1 + Stimulus_deg_A * Stimulus_deg_V | Participant)
##    Data: Z_DfV
## 
## REML criterion at convergence: 9959.4
## 
## Scaled residuals: 
##     Min      1Q  Median      3Q     Max 
## -3.4139 -0.5696 -0.0377  0.5960  3.5382 
## 
## Random effects:
##  Groups          Name                          Variance Std.Dev. Corr       
##  Participant     (Intercept)                   417.9658 20.4442             
##                  Stimulus_deg_A                  1.9145  1.3837   0.81      
##                  Stimulus_deg_V                 16.9522  4.1173   0.16 -0.46
##                  Stimulus_deg_A:Stimulus_deg_V   1.0933  1.0456   0.53 -0.07
##  Stimulus_talker (Intercept)                     4.0963  2.0239             
##                  Stimulus_deg_A                  2.1268  1.4584  -0.94      
##                  Stimulus_deg_V                  1.3633  1.1676   0.38 -0.66
##                  Stimulus_deg_A:Stimulus_deg_V   0.3382  0.5816  -0.97  0.89
##  Residual                                      390.3512 19.7573             
##       
##       
##       
##       
##   0.92
##       
##       
##       
##  -0.35
##       
## Number of obs: 1116, groups:  Participant, 31; Stimulus_talker, 4
## 
## Fixed effects:
##                               Estimate Std. Error       df t value Pr(>|t|)  
## (Intercept)                   20.21672   11.15974 30.83732   1.812   0.0798 .
## Fixation_cross                 8.10522    6.90770 29.65435   1.173   0.2500  
## Stimulus_deg_A                 1.24879    0.97137  3.88488   1.286   0.2699  
## Stimulus_deg_V                -2.61682    1.11255  8.61125  -2.352   0.0444 *
## Stimulus_deg_A:Stimulus_deg_V  0.05865    0.68573  4.12718   0.086   0.9358  
## ---
## Signif. codes:  0 '***' 0.001 '**' 0.01 '*' 0.05 '.' 0.1 ' ' 1
## 
## Correlation of Fixed Effects:
##             (Intr) Fxtn_c Stm__A Stm__V
## Fixatn_crss -0.938                     
## Stimls_dg_A  0.004  0.000              
## Stimls_dg_V  0.052  0.000 -0.339       
## Stm__A:S__V  0.011  0.000  0.279  0.089
## convergence code: 0
## boundary (singular) fit: see ?isSingular
```

#compare models

```
anova(Model7V,Model6V)
```

```
## refitting model(s) with ML (instead of REML)
```

```
## Data: Z_DfV
## Models:
## Model6V: Dwell_time ~ Fixation_cross + Stimulus_deg_A + Stimulus_deg_V + 
## Model6V:     (1 + Stimulus_deg_A * Stimulus_deg_V | Stimulus_talker) + 
## Model6V:     (1 + Stimulus_deg_A * Stimulus_deg_V | Participant)
## Model7V: Dwell_time ~ Fixation_cross + Stimulus_deg_A * Stimulus_deg_V + 
## Model7V:     (1 + Stimulus_deg_A * Stimulus_deg_V | Stimulus_talker) + 
## Model7V:     (1 + Stimulus_deg_A * Stimulus_deg_V | Participant)
##         Df   AIC   BIC logLik deviance  Chisq Chi Df Pr(>Chisq)
## Model6V 25 10024 10149  -4987     9974                         
## Model7V 26 10026 10156  -4987     9974 0.0079      1      0.929
```

#can drop interaction, use model6V but now we are overfitting, dropping interaction of random effect talker due to low variance

```
Model8V<- lmer(Dwell_time ~ Fixation_cross + Stimulus_deg_A+Stimulus_deg_V + (1+Stimulus_deg_A+Stimulus_deg_V|Stimulus_talker)+(1+Stimulus_deg_A*Stimulus_deg_V|Participant), data=Z_DfV, REML = TRUE)
```

```
## boundary (singular) fit: see ?isSingular
```

```
summary(Model8V)
```

```
## Linear mixed model fit by REML. t-tests use Satterthwaite's method [
## lmerModLmerTest]
## Formula: Dwell_time ~ Fixation_cross + Stimulus_deg_A + Stimulus_deg_V +  
##     (1 + Stimulus_deg_A + Stimulus_deg_V | Stimulus_talker) +  
##     (1 + Stimulus_deg_A * Stimulus_deg_V | Participant)
##    Data: Z_DfV
## 
## REML criterion at convergence: 9960.8
## 
## Scaled residuals: 
##     Min      1Q  Median      3Q     Max 
## -3.4244 -0.5465 -0.0423  0.6014  3.5517 
## 
## Random effects:
##  Groups          Name                          Variance Std.Dev. Corr       
##  Participant     (Intercept)                   417.516  20.433              
##                  Stimulus_deg_A                  1.915   1.384    0.81      
##                  Stimulus_deg_V                 16.904   4.111    0.15 -0.46
##                  Stimulus_deg_A:Stimulus_deg_V   1.008   1.004    0.53 -0.07
##  Stimulus_talker (Intercept)                     4.031   2.008              
##                  Stimulus_deg_A                  2.210   1.487   -0.95      
##                  Stimulus_deg_V                  1.366   1.169    0.37 -0.64
##  Residual                                      390.311  19.756              
##       
##       
##       
##       
##   0.92
##       
##       
##       
##       
## Number of obs: 1116, groups:  Participant, 31; Stimulus_talker, 4
## 
## Fixed effects:
##                Estimate Std. Error     df t value Pr(>|t|)  
## (Intercept)      20.159     11.146 30.684   1.809   0.0803 .
## Fixation_cross    8.108      6.909 29.660   1.173   0.2499  
## Stimulus_deg_A    1.249      0.982  3.865   1.272   0.2744  
## Stimulus_deg_V   -2.635      1.095  8.236  -2.408   0.0418 *
## ---
## Signif. codes:  0 '***' 0.001 '**' 0.01 '*' 0.05 '.' 0.1 ' ' 1
## 
## Correlation of Fixed Effects:
##             (Intr) Fxtn_c Stm__A
## Fixatn_crss -0.940              
## Stimls_dg_A  0.003  0.000       
## Stimls_dg_V  0.043  0.000 -0.337
## convergence code: 0
## boundary (singular) fit: see ?isSingular
```

#still overfitting, removing high correlations

```
Model9V<- lmer(Dwell_time ~ Fixation_cross + Stimulus_deg_A+Stimulus_deg_V + (0+Stimulus_deg_A|Stimulus_talker)+ (1+Stimulus_deg_V|Stimulus_talker)+(0+Stimulus_deg_A|Participant)+ (1+Stimulus_deg_V|Participant), data=Z_DfV, REML = TRUE)
```

```
## Warning in checkConv(attr(opt, "derivs"), opt$par, ctrl = control$checkConv, :
## Model failed to converge with max|grad| = 0.0091033 (tol = 0.002, component 1)
```

```
summary(Model9V)
```

```
## Linear mixed model fit by REML. t-tests use Satterthwaite's method [
## lmerModLmerTest]
## Formula: Dwell_time ~ Fixation_cross + Stimulus_deg_A + Stimulus_deg_V +  
##     (0 + Stimulus_deg_A | Stimulus_talker) + (1 + Stimulus_deg_V |  
##     Stimulus_talker) + (0 + Stimulus_deg_A | Participant) + (1 +  
##     Stimulus_deg_V | Participant)
##    Data: Z_DfV
## 
## REML criterion at convergence: 9971.2
## 
## Scaled residuals: 
##     Min      1Q  Median      3Q     Max 
## -3.3474 -0.5627 -0.0507  0.6405  3.5357 
## 
## Random effects:
##  Groups            Name           Variance  Std.Dev. Corr
##  Participant       (Intercept)    4.168e+02 20.4161      
##                    Stimulus_deg_V 1.640e+01  4.0501  0.16
##  Participant.1     Stimulus_deg_A 1.878e-04  0.0137      
##  Stimulus_talker   (Intercept)    3.715e+00  1.9274      
##                    Stimulus_deg_V 1.186e+00  1.0892  0.32
##  Stimulus_talker.1 Stimulus_deg_A 1.396e+00  1.1816      
##  Residual                         3.948e+02 19.8690      
## Number of obs: 1116, groups:  Participant, 31; Stimulus_talker, 4
## 
## Fixed effects:
##                Estimate Std. Error      df t value Pr(>|t|)  
## (Intercept)     25.4538    11.8292 29.4525   2.152   0.0397 *
## Fixation_cross   4.6510     7.3803 29.0122   0.630   0.5335  
## Stimulus_deg_A   1.2488     0.8385  2.9961   1.489   0.2333  
## Stimulus_deg_V  -2.6168     1.0862  8.3522  -2.409   0.0413 *
## ---
## Signif. codes:  0 '***' 0.001 '**' 0.01 '*' 0.05 '.' 0.1 ' ' 1
## 
## Correlation of Fixed Effects:
##             (Intr) Fxtn_c Stm__A
## Fixatn_crss -0.946              
## Stimls_dg_A  0.000  0.000       
## Stimls_dg_V  0.045  0.000  0.000
## convergence code: 0
## Model failed to converge with max|grad| = 0.0091033 (tol = 0.002, component 1)
```

#drop all correlations to solve convergence issues - doesn’t work

```
Model10V<- lmer(Dwell_time ~ Fixation_cross + Stimulus_deg_A+Stimulus_deg_V + (1+Stimulus_deg_V+Stimulus_deg_A||Stimulus_talker)+ (1+Stimulus_deg_A+Stimulus_deg_V||Participant), data=Z_DfV)
```

```
## Warning in checkConv(attr(opt, "derivs"), opt$par, ctrl = control$checkConv, :
## Model failed to converge with max|grad| = 0.0024341 (tol = 0.002, component 1)
```

```
summary(Model10V)
```

```
## Linear mixed model fit by REML. t-tests use Satterthwaite's method [
## lmerModLmerTest]
## Formula: Dwell_time ~ Fixation_cross + Stimulus_deg_A + Stimulus_deg_V +  
##     (1 + Stimulus_deg_V + Stimulus_deg_A || Stimulus_talker) +  
##     (1 + Stimulus_deg_A + Stimulus_deg_V || Participant)
##    Data: Z_DfV
## 
## REML criterion at convergence: 9971.7
## 
## Scaled residuals: 
##     Min      1Q  Median      3Q     Max 
## -3.3154 -0.5598 -0.0576  0.6331  3.5300 
## 
## Random effects:
##  Groups            Name           Variance  Std.Dev. 
##  Participant       Stimulus_deg_V 1.640e+01  4.049424
##  Participant.1     Stimulus_deg_A 1.204e-05  0.003469
##  Participant.2     (Intercept)    4.173e+02 20.428194
##  Stimulus_talker   Stimulus_deg_A 1.391e+00  1.179222
##  Stimulus_talker.1 Stimulus_deg_V 1.198e+00  1.094318
##  Stimulus_talker.2 (Intercept)    3.712e+00  1.926767
##  Residual                         3.948e+02 19.868815
## Number of obs: 1116, groups:  Participant, 31; Stimulus_talker, 4
## 
## Fixed effects:
##                Estimate Std. Error      df t value Pr(>|t|)  
## (Intercept)     25.6427    11.9123 29.3455   2.153   0.0397 *
## Fixation_cross   4.5264     7.4377 28.9861   0.609   0.5475  
## Stimulus_deg_A   1.2488     0.8377  3.0020   1.491   0.2327  
## Stimulus_deg_V  -2.6168     1.0874  8.3042  -2.407   0.0417 *
## ---
## Signif. codes:  0 '***' 0.001 '**' 0.01 '*' 0.05 '.' 0.1 ' ' 1
## 
## Correlation of Fixed Effects:
##             (Intr) Fxtn_c Stm__A
## Fixatn_crss -0.947              
## Stimls_dg_A  0.000  0.000       
## Stimls_dg_V  0.000  0.000  0.000
## convergence code: 0
## Model failed to converge with max|grad| = 0.0024341 (tol = 0.002, component 1)
```

#change optimizer to solve convergence issues - doesn’t work

```
Model11V<- lmer(Dwell_time ~ Fixation_cross + Stimulus_deg_A+Stimulus_deg_V + (0+Stimulus_deg_A|Stimulus_talker)+ (1+Stimulus_deg_V|Stimulus_talker)+(0+Stimulus_deg_A|Participant)+ (1+Stimulus_deg_V|Participant), data=Z_DfV, control = lmerControl(optimizer = "Nelder_Mead"))
```

```
## Warning in checkConv(attr(opt, "derivs"), opt$par, ctrl = control$checkConv, :
## unable to evaluate scaled gradient
```

```
## Warning in checkConv(attr(opt, "derivs"), opt$par, ctrl = control$checkConv, :
## Model failed to converge: degenerate Hessian with 1 negative eigenvalues
```

```
## Warning: Model failed to converge with 1 negative eigenvalue: -3.0e+00
```

```
summary(Model11V)
```

```
## Linear mixed model fit by REML. t-tests use Satterthwaite's method [
## lmerModLmerTest]
## Formula: Dwell_time ~ Fixation_cross + Stimulus_deg_A + Stimulus_deg_V +  
##     (0 + Stimulus_deg_A | Stimulus_talker) + (1 + Stimulus_deg_V |  
##     Stimulus_talker) + (0 + Stimulus_deg_A | Participant) + (1 +  
##     Stimulus_deg_V | Participant)
##    Data: Z_DfV
## Control: lmerControl(optimizer = "Nelder_Mead")
## 
## REML criterion at convergence: 9985.1
## 
## Scaled residuals: 
##     Min      1Q  Median      3Q     Max 
## -3.3496 -0.5447 -0.0563  0.6395  3.4873 
## 
## Random effects:
##  Groups            Name           Variance Std.Dev. Corr
##  Participant       (Intercept)    401.7963 20.0449      
##                    Stimulus_deg_V  16.4864  4.0604  0.16
##  Participant.1     Stimulus_deg_A   0.2129  0.4614      
##  Stimulus_talker   (Intercept)      3.6660  1.9147      
##                    Stimulus_deg_V   1.2971  1.1389  0.30
##  Stimulus_talker.1 Stimulus_deg_A 765.0658 27.6598      
##  Residual                         393.7890 19.8441      
## Number of obs: 1116, groups:  Participant, 31; Stimulus_talker, 4
## 
## Fixed effects:
##                Estimate Std. Error       df t value Pr(>|t|)  
## (Intercept)      25.458     11.621   31.052   2.191   0.0361 *
## Fixation_cross    4.648      7.250   30.580   0.641   0.5262  
## Stimulus_deg_A    1.249     13.843 1036.803   0.090   0.9281  
## Stimulus_deg_V   -2.617      1.100    7.949  -2.380   0.0448 *
## ---
## Signif. codes:  0 '***' 0.001 '**' 0.01 '*' 0.05 '.' 0.1 ' ' 1
## 
## Correlation of Fixed Effects:
##             (Intr) Fxtn_c Stm__A
## Fixatn_crss -0.946              
## Stimls_dg_A  0.000  0.000       
## Stimls_dg_V  0.045  0.000  0.000
## convergence code: 0
## unable to evaluate scaled gradient
## Model failed to converge: degenerate  Hessian with 1 negative eigenvalues
```

```
relgrad <- with(Model9V@optinfo$derivs,solve(Hessian,gradient))
max(abs(relgrad))
```

```
## [1] 0.0006893358
```

#can ignore convergence warning for Model9V, report

## 3. Vocoded speech: Association between McGUrk perception and dwell time on mouth, according to fixation cross position

#full model with interaction

```
Model12V <- lmer(Dwell_time ~ Fixation_cross * Accuracy + (1+Accuracy|Participant) + (1+Accuracy|Stimulus_talker), data=Z_DfV, REML = TRUE)
```

```
## Warning in checkConv(attr(opt, "derivs"), opt$par, ctrl = control$checkConv, :
## Model failed to converge with max|grad| = 0.00938494 (tol = 0.002, component 1)
```

```
summary(Model12V)
```

```
## Linear mixed model fit by REML. t-tests use Satterthwaite's method [
## lmerModLmerTest]
## Formula: 
## Dwell_time ~ Fixation_cross * Accuracy + (1 + Accuracy | Participant) +  
##     (1 + Accuracy | Stimulus_talker)
##    Data: Z_DfV
## 
## REML criterion at convergence: 10000.5
## 
## Scaled residuals: 
##     Min      1Q  Median      3Q     Max 
## -3.3979 -0.5642 -0.0570  0.6110  3.6334 
## 
## Random effects:
##  Groups          Name        Variance Std.Dev. Corr 
##  Participant     (Intercept) 439.8556 20.9727       
##                  Accuracy      0.5293  0.7275  -1.00
##  Stimulus_talker (Intercept)   0.1887  0.4343       
##                  Accuracy      5.9622  2.4418  1.00 
##  Residual                    418.7120 20.4625       
## Number of obs: 1116, groups:  Participant, 31; Stimulus_talker, 4
## 
## Fixed effects:
##                         Estimate Std. Error       df t value Pr(>|t|)  
## (Intercept)              26.2000    12.6071  29.1195   2.078   0.0466 *
## Fixation_cross            2.9846     7.9096  29.3016   0.377   0.7086  
## Accuracy                 -0.4977     4.6440 110.4141  -0.107   0.9149  
## Fixation_cross:Accuracy   2.0970     2.8375 623.0317   0.739   0.4602  
## ---
## Signif. codes:  0 '***' 0.001 '**' 0.01 '*' 0.05 '.' 0.1 ' ' 1
## 
## Correlation of Fixed Effects:
##             (Intr) Fxtn_c Accrcy
## Fixatn_crss -0.949              
## Accuracy    -0.325  0.317       
## Fxtn_crss:A  0.325 -0.347 -0.915
## convergence code: 0
## Model failed to converge with max|grad| = 0.00938494 (tol = 0.002, component 1)
```

#model without interaction

```
Model13V <- lmer(Dwell_time ~ Fixation_cross + Accuracy + (1+Accuracy|Participant) + (1+Accuracy|Stimulus_talker), data=Z_DfV, REML=TRUE)
```

```
## boundary (singular) fit: see ?isSingular
```

```
## Warning: Model failed to converge with 1 negative eigenvalue: -2.1e-01
```

```
summary(Model13V)
```

```
## Linear mixed model fit by REML. t-tests use Satterthwaite's method [
## lmerModLmerTest]
## Formula: 
## Dwell_time ~ Fixation_cross + Accuracy + (1 + Accuracy | Participant) +  
##     (1 + Accuracy | Stimulus_talker)
##    Data: Z_DfV
## 
## REML criterion at convergence: 10005
## 
## Scaled residuals: 
##     Min      1Q  Median      3Q     Max 
## -3.4161 -0.5574 -0.0586  0.6261  3.6398 
## 
## Random effects:
##  Groups          Name        Variance Std.Dev. Corr 
##  Participant     (Intercept) 439.0401 20.9533       
##                  Accuracy      0.4952  0.7037  -1.00
##  Stimulus_talker (Intercept)   0.1144  0.3383       
##                  Accuracy      6.4202  2.5338  0.96 
##  Residual                    418.5497 20.4585       
## Number of obs: 1116, groups:  Participant, 31; Stimulus_talker, 4
## 
## Fixed effects:
##                Estimate Std. Error     df t value Pr(>|t|)  
## (Intercept)      23.226     11.922 29.631   1.948   0.0609 .
## Fixation_cross    4.999      7.419 28.939   0.674   0.5058  
## Accuracy          2.609      1.907  3.511   1.368   0.2523  
## ---
## Signif. codes:  0 '***' 0.001 '**' 0.01 '*' 0.05 '.' 0.1 ' ' 1
## 
## Correlation of Fixed Effects:
##             (Intr) Fxtn_c
## Fixatn_crss -0.943       
## Accuracy    -0.074 -0.002
## convergence code: 0
## boundary (singular) fit: see ?isSingular
```

#compare models

```
anova(Model12V,Model13V)
```

```
## refitting model(s) with ML (instead of REML)
```

```
## Data: Z_DfV
## Models:
## Model13V: Dwell_time ~ Fixation_cross + Accuracy + (1 + Accuracy | Participant) + 
## Model13V:     (1 + Accuracy | Stimulus_talker)
## Model12V: Dwell_time ~ Fixation_cross * Accuracy + (1 + Accuracy | Participant) + 
## Model12V:     (1 + Accuracy | Stimulus_talker)
##          Df   AIC   BIC  logLik deviance  Chisq Chi Df Pr(>Chisq)
## Model13V 10 10038 10088 -5009.1    10018                         
## Model12V 11 10040 10095 -5008.9    10018 0.5683      1     0.4509
```

#can drop interaction, model 13 failed to converge, dropping random effects with low variance and removing high correlations

```
Model14V <- lmer(Dwell_time ~ Fixation_cross + Accuracy + (1|Participant) + (0+Accuracy|Stimulus_talker)+(1|Stimulus_talker), data=Z_DfV, REML=TRUE)
summary(Model14V)
```

```
## Linear mixed model fit by REML. t-tests use Satterthwaite's method [
## lmerModLmerTest]
## Formula: Dwell_time ~ Fixation_cross + Accuracy + (1 | Participant) +  
##     (0 + Accuracy | Stimulus_talker) + (1 | Stimulus_talker)
##    Data: Z_DfV
## 
## REML criterion at convergence: 10005.2
## 
## Scaled residuals: 
##     Min      1Q  Median      3Q     Max 
## -3.4357 -0.5518 -0.0619  0.6262  3.6408 
## 
## Random effects:
##  Groups            Name        Variance Std.Dev.
##  Participant       (Intercept) 418.172  20.4493 
##  Stimulus_talker   Accuracy      7.791   2.7912 
##  Stimulus_talker.1 (Intercept)   0.349   0.5908 
##  Residual                      418.593  20.4595 
## Number of obs: 1116, groups:  Participant, 31; Stimulus_talker, 4
## 
## Fixed effects:
##                Estimate Std. Error     df t value Pr(>|t|)  
## (Intercept)      24.064     11.937 29.411   2.016    0.053 .
## Fixation_cross    4.522      7.451 29.016   0.607    0.549  
## Accuracy          2.497      1.993  6.952   1.253    0.251  
## ---
## Signif. codes:  0 '***' 0.001 '**' 0.01 '*' 0.05 '.' 0.1 ' ' 1
## 
## Correlation of Fixed Effects:
##             (Intr) Fxtn_c
## Fixatn_crss -0.946       
## Accuracy    -0.057 -0.005
```

#Report model 14V #End
